# Bioactive compounds from Perilla frutescens modulate multiple cell death pathways and signaling networks in cancer therapy

**DOI:** 10.3389/fphar.2026.1956245

**Published:** 2026-09-28

**Authors:** Hai Xu, Jingyi Liu, Huikun Wu, Hussein Sabit, Bangquan Huang, Yong Zhou, Lei Wang, Linhai Wang

**Affiliations:** 1 Huangjiahu Hospital, Hubei University of Chinese Medicine, Wuhan, China; 2 Hubei Province International Science and Technology Cooperation Base for Innovative Research and Development of Food and Drug Resources and Targeted Delivery, Wuhan, China; 3 Hubei Shizhen Laboratory, Hubei University of Chinese Medicine, Wuhan, China; 4 First Clinical College, Hubei University of Chinese Medicine, Wuhan, China; 5 Department of Medical Biotechnology, College of Biotechnology, Misr University for Science and Technology, Giza, Egypt; 6 College of Life Science, Hubei University, Wuhan, China; 7 Oncology Department, Hubei Provincial Hospital of Traditional Chinese Medicine, Wuhan, China; 8 Key Laboratory of Biology and Genetic Improvement of Oil Crops, Ministry of Agriculture and Rural Affairs, Oil Crops Research Institute, Chinese Academy of Agricultural Sciences, Wuhan, China

**Keywords:** apoptosis, autophagy, luteolin, Perilla frutescens, perillaldehyde, rosmarinic acid

## Abstract

Perilla frutescens, an edible medicinal plant from East Asia, produces diverse bioactive phytochemicals with anticancer activity, including perillaldehyde (PAH), rosmarinic acid (RA), luteolin, apigenin, and alpha-linolenic acid (ALA). These compounds exert multi-target anticancer effects through apoptosis, autophagy, and ferroptosis. PAH triggers ferroptosis in gastric cancer via the System Xc-/GSH/GPX4 axis and P62-Keap1-Nrf2 pathway, while inducing protective autophagy in colorectal cancer through SRD5A1 and PI3K/AKT modulation. RA directly binds KRAS(G12C) and suppresses NSCLC via KRAS/AKT/ERK, and inhibits stress-induced TNBC metastasis through Src inactivation. *Perilla* leaf extracts activate Hippo-YAP/TAZ signaling in breast cancer. ALA-rich perilla seed oil (PSO) mitigates tumorigenesis in colitis-associated colorectal cancer models and restores gut microbial homeostasis. This review synthesizes current molecular evidence on perilla compounds in cancer therapy, focusing on apoptosis-autophagy-ferroptosis crosstalk. The multi-target characteristics position perilla-derived compounds as adjuvant cancer therapy candidates, though bioavailability and clinical translation still challenges remain.

## Graphical Abstract

**Figure:**
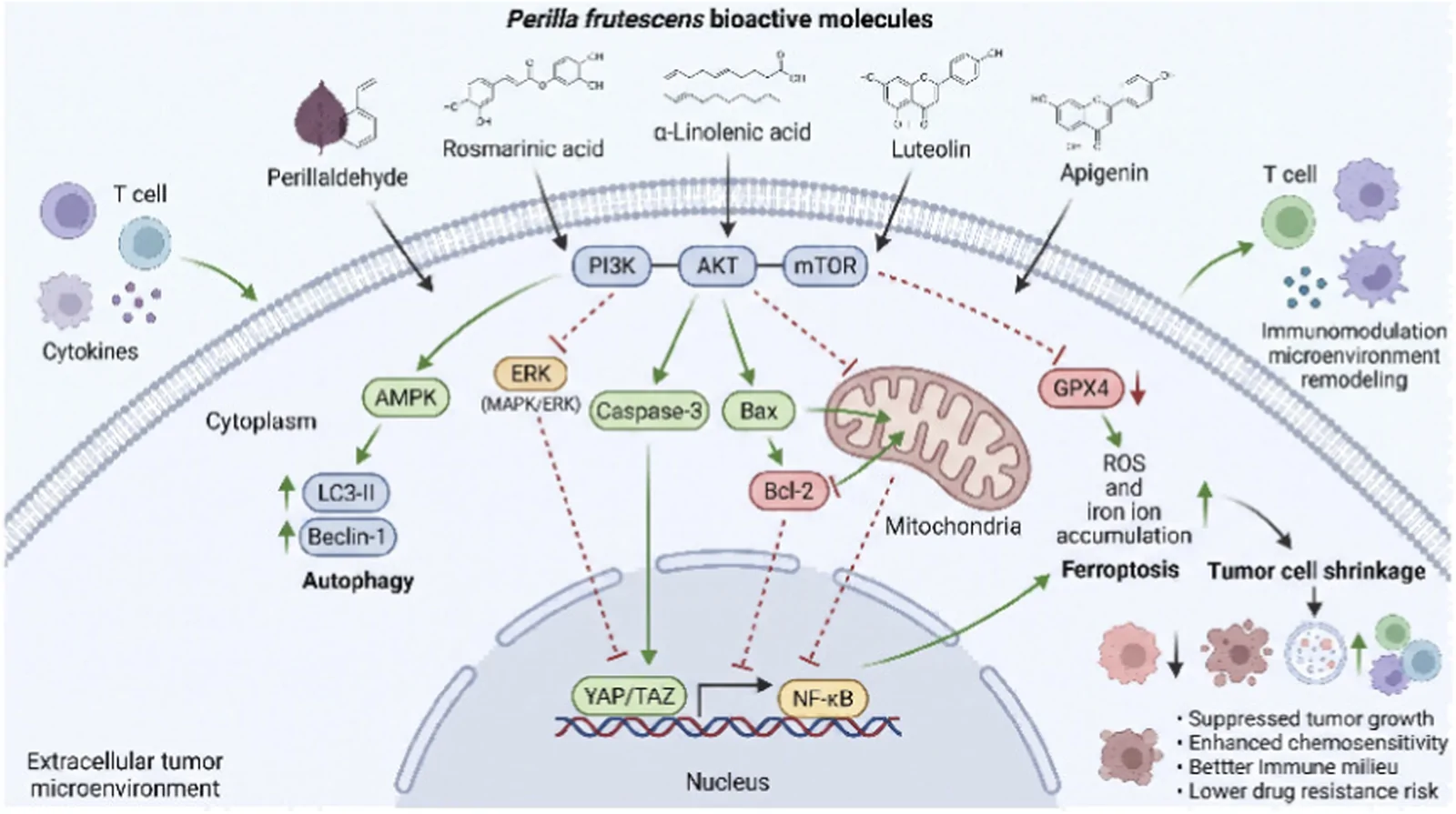
Bioactive compounds from *Perilla frutescens* such as PAH, RA, ALA, luteolin, apigenin exert anticancer activity through coordinated induction of apoptosis, autophagy, and ferroptosis, modulation of PI3K/AKT, AMPK, Hippo, MAPK, and NF-κB signaling, and reversal of chemoresistance. This multi-target mechanism positions *Perilla* phytochemicals as promising candidates for adjuvant cancer therapy.

## 1 Introduction

### 1.1 *Perilla frutescens*: from traditional medicine to modern drug discovery

Perilla frutescens (L.) is an annual herbaceous plant (Lamiaceae) cultivated for centuries across East Asia. All aerial parts—leaves (Perillae Folium), stems (Perillae Caulis), and seeds (Perillae Fructus)—are used in traditional Chinese medicine. The Compendium of Materia Medica documented perilla for treating respiratory, gastrointestinal, and inflammatory conditions. Modern studies have validated these applications, revealing antioxidant, anti-inflammatory, antibacterial, and hepatoprotective activities.

Over 100 bioactive constituents have been identified from perilla, including volatile monoterpenoids (perillaldehyde, limonene, beta-caryophyllene), polyphenolic acids (rosmarinic, caffeic, ferulic acid), flavonoids (luteolin, apigenin, quercetin), and polyunsaturated fatty acids (ALA). These compounds inhibit a broad spectrum of cancers—gastric, colorectal, breast, lung, liver, and hematological malignancies—through diverse molecular mechanisms reviewed herein.

### 1.2 Challenges in contemporary cancer therapy

Despite significant advancements in cancer treatment over the past several decades, including targeted therapies, immunotherapies, and precision medicine approaches, cancer remains the second leading cause of death worldwide (Bray et al., 2024). Conventional chemotherapeutic agents, while effective in many cases, are frequently associated with severe adverse effects, high toxicity to normal tissues, and the emergence of multidrug resistance (MDR) that ultimately leads to treatment failure (Holohan et al., 2013). The development of resistance—driven by genetic mutations, epigenetic adaptations, and tumor heterogeneity—is a formidable obstacle that limits the long-term efficacy of both conventional chemotherapy and molecularly targeted agents.

Natural products offer therapeutic advantages: pleiotropic multi-pathway modulation reduces resistance development, exhibits a degree of selective cytotoxicity toward cancer cells while sparing normal tissues, and synergistically enhances conventional chemotherapy while mitigating side effects. Among many traditional medicinal plants, *Perilla frutescens* stands out for its favorable safety profile and structurally diverse bioactive constituents (Pandit et al., 2025).

### 1.3 Gaps in the current literature

The sole comprehensive review on perilla anticancer mechanisms was published by Huang et al. (2023), providing foundational chemical and pathway overviews. However, several critical advances since then were not covered: (i) ferroptosis induction by perillaldehyde in gastric cancer and AML (Wang et al., 2025; Catanzaro et al., 2022); (ii) novel molecular targets including KRAS(G12C) by rosmarinic acid, HSP70 in CML (Zhang et al., 2025), and Hippo-YAP/TAZ by perilla leaf extracts; (iii) the therapeutic potential of perilla seed oil (PSO) and its effects on the tumor microenvironment (TME) and gut microbiome; (iv) systematic analysis of crosstalk among apoptosis, autophagy, and ferroptosis signaling networks; and (v) emerging strategies including nanoformulation and combination therapy approaches for clinical translation.

### 1.4 Scope and structure of this review

Figure 1 provides an integrated overview of the signaling networks and cell death pathways modulated by Perilla frutescens-derived compounds. This review is organized as follows: Section 2 describes bioactive constituents and their chemical structures; Section 3 examines regulation of apoptosis, autophagy, and ferroptosis by perilla compounds; Section 4 focuses on key signaling pathway modulation; Section 5 discusses cancer type-specific effects; Section 6 highlights emerging strategies (nanotechnology, combination therapy); and Section 7 addresses challenges and future directions for clinical translation, integrating findings from 2023 to 2026.

**FIGURE 1:**
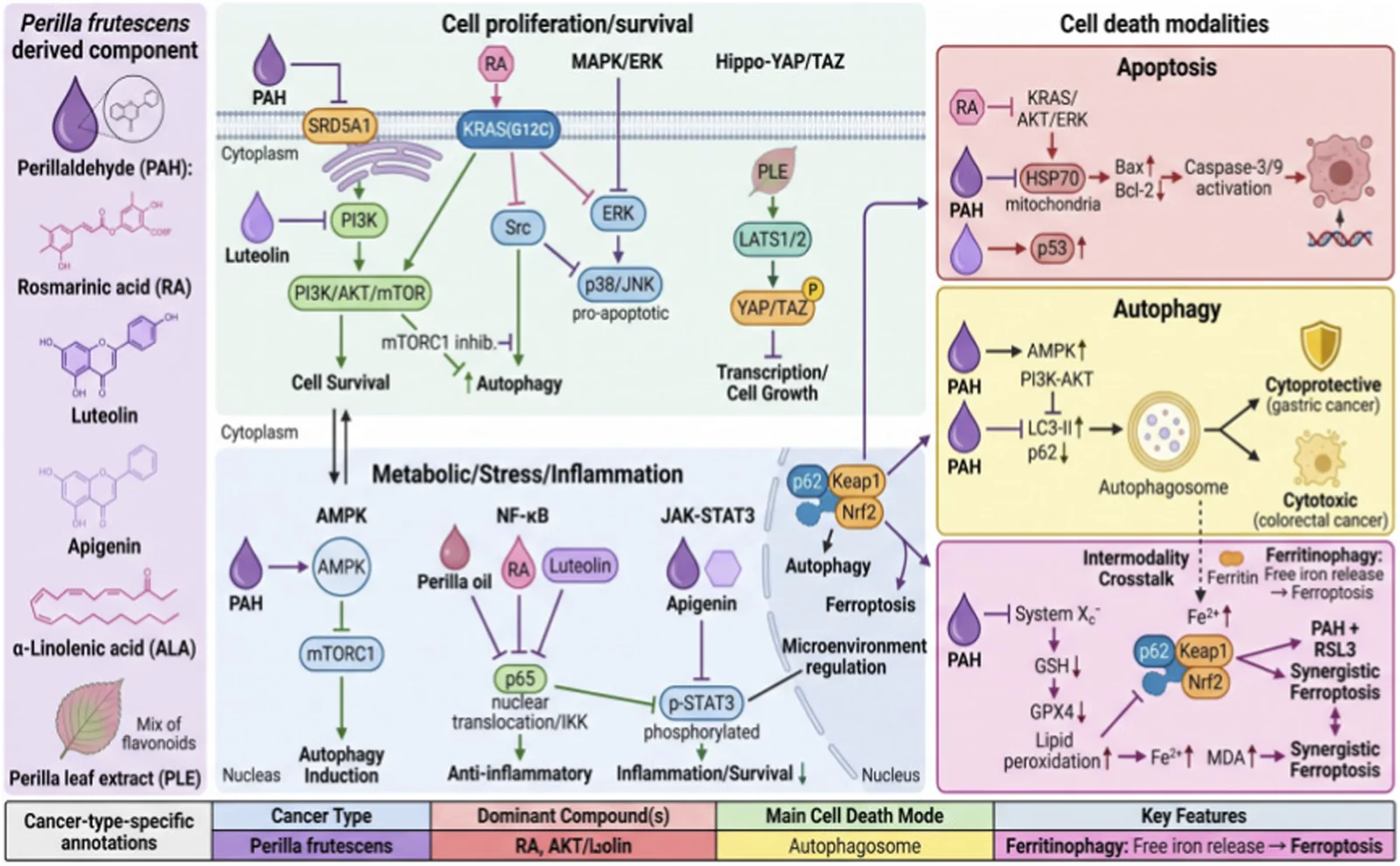
Integrative mechanistic network of Perilla frutescens-derived compounds regulating apoptosis, autophagy, and ferroptosis in cancer.

## 2 Bioactive constituents of *Perilla frutescens*

*Perilla frutescens* is a rich source of structurally diverse secondary metabolites with documented anticancer activities. The bioactive compounds can be broadly categorized into volatile oils (terpenoids), phenolic acids, flavonoids, and polyunsaturated fatty acids. The chemical diversity of these constituents enables the pleiotropic pharmacological effects observed in preclinical cancer models. This section provides a systematic overview of the major bioactive compounds, their chemical characteristics, plant sources, and reported anticancer activities.

### 2.1 Volatile oils (terpenoids)

The essential oil of *Perilla frutescens*, predominantly extracted from the leaves, contains a complex mixture of monoterpenoids and sesquiterpenoids. The most abundant and biologically significant volatile compound is perillaldehyde (PAH, also known as perillaldehyde or perilla aldehyde), a monoterpene with the molecular formula C10H14O. PAH typically constitutes 50%–70% of perilla essential oil and is responsible for the characteristic perilla aroma (Eltokhy et al., 2026). It is biosynthesized through the mevalonate pathway and is classified as a food-grade flavoring agent by the U.S. Food and Drug Administration (FDA), highlighting its safety profile.

Accumulating evidence demonstrates that PAH possesses broad-spectrum anticancer activities. It has been shown to inhibit colorectal cancer growth by targeting SRD5A1 and inducing autophagy via the PI3K/AKT pathway, trigger ferroptosis in gastric cancer through the System Xc-/GSH/GPX4 axis and P62-Keap1-Nrf2 pathway, overcome tyrosine kinase inhibitor resistance in chronic myeloid leukemia by targeting HSP70, and suppress prostate cancer bone metastasis through inhibition of RANKL signaling-induced osteoclastogenesis (Lin et al., 2022). Additionally, PAH activates AMP-activated protein kinase (AMPK) to induce autophagy-dependent cell death in gastric cancer cells (Zhang et al., 2019).

Other notable perilla volatile compounds include limonene, a monocyclic monoterpene with documented chemopreventive properties, and beta-caryophyllene, a bicyclic sesquiterpene that exhibits anti-inflammatory and anticancer activities through cannabinoid receptor type 2 (CB2) activation. Isoegomaketone, a furan-containing monoterpenoid unique to Perilla species, has recently been reported to possess anti-inflammatory activity that may contribute to its anticancer potential (Choi et al., 2025).

### 2.2 Phenolic acids

Rosmarinic acid (RA, C_18_H_16_O_8_), an ester of caffeic and 3,4-dihydroxyphenyllactic acid, is the predominant phenolic acid from perilla leaves and seeds (Leong et al., 2026). RA exhibits potential antioxidant, anti-inflammatory, and anticancer properties. Zhao et al. (Zhao et al., 2026) demonstrated that RA directly binds KRAS(G12C), suppressing NSCLC progression via KRAS/AKT/ERK modulation. Jeong et al. (Jeong et al., 2026) reported that RA inhibits norepinephrine-induced TNBC metastasis through Src kinase inactivation. Additionally, RA protects against chemotherapy-induced nephrotoxicity by disrupting IL-17A-ferroptosis coupling (Süzen Celbek et al., 2026). Other phenolic acids (caffeic, ferulic) contribute to perilla’s antioxidant and anticancer properties through free radical scavenging and modulation of Nrf2 and NF-κB.

Other phenolic acids in perilla include caffeic acid and ferulic acid, which contribute to the overall antioxidant and anticancer properties of perilla extracts. These compounds act primarily through free radical scavenging, modulation of redox-sensitive transcription factors (e.g., Nrf2, NF-kappaB), and inhibition of cancer cell proliferation.

### 2.3 Flavonoids

Perilla leaves are rich in flavonoids, with luteolin, apigenin, and quercetin being the most abundant. Luteolin (C_15_H_10_O_6_), a tetrahydroxyflavone, is one of the most widely studied flavonoids for its anticancer properties (Hostetler et al., 2017). It modulates multiple signaling pathways including NF-kappaB, MAPK, PI3K/AKT, and JAK-STAT (Binmahfouz, 2026). Using network pharmacology combined with transcriptome sequencing, Han et al. (Han et al., 2025) identified key molecular targets of luteolin in hepatocellular carcinoma, revealing its multi-target mechanism of action (Han et al., 2025). Luteolin also exerts regulatory effects on redox balance and inflammation, which are critical for cancer prevention and treatment. Luteolin exhibits low solubility and high permeability, with an absolute bioavailability of only 3.87%–5.57% in rats.

Apigenin (C_15_H_10_O_5_) is structurally similar to luteolin and shares many of its anticancer mechanisms, including cell cycle arrest, apoptosis induction, and inhibition of metastasis. Quercetin, a flavonol, adds to the antioxidant capacity of perilla extracts. Notably, Kim et al. (Kim et al., 2021) demonstrated that the flavonoid-rich ethanol extract of perilla leaves exhibits antitumor effects on breast cancer cells by suppressing YAP/TAZ transcriptional activity through the Hippo signaling pathway, an effect likely mediated by the combined action of multiple flavonoids present in the extract.

### 2.4 Polyunsaturated fatty acids from perilla seed oil

PSO is one of the richest plant sources of omega-3 polyunsaturated fatty acids, containing approximately 50%–60% alpha-linolenic acid (ALA, C_18_H_30_O_2_; 18:3, n-3), along with linoleic acid (LA, 18:2, n-6) and oleic acid (18:1, n-9) (Korsirikoon et al., 2024). ALA, as the parent compound of the omega-3 fatty acid family, serves as a precursor for the biosynthesis of eicosapentaenoic acid (EPA) and docosahexaenoic acid (DHA), though the conversion rate is limited in humans.

The anticancer effects of PSO and ALA have been demonstrated in several experimental models (Calder, 2017). Korsirikoon et al. (Korsirikoon et al., 2024) showed that cold-pressed PSO enriched with ALA significantly mitigated tumor progression in the AOM/DSS mouse model of colitis-associated colorectal cancer and restored gut microbial homeostasis. The mechanisms involve modulation of inflammation, lipid peroxidation, and alteration of the gut microbiota composition. Previous studies have also established that perilla oil suppresses NF-kappaB activation in the colon and reduces proinflammatory cytokine production (Thomas et al., 2020), providing a mechanistic basis for its chemopreventive effects against colorectal carcinogenesis.

Alpha-linolenic acid (ALA, C18:3 n-3), the predominant fatty acid in PSO, serves as the metabolic precursor for a cascade of biologically active long-chain polyunsaturated fatty acids. Through sequential desaturation and elongation catalyzed by delta-6 desaturase (FADS2), elongase (ELOVL5), and delta-5 desaturase (FADS1), ALA is converted to eicosapentaenoic acid (EPA, C20:5 n-3) and subsequently to docosahexaenoic acid (DHA, C22:6 n-3) (Calder, 2017). The conversion efficiency in humans is limited (approximately 5%–10% for EPA and 2%–5% for DHA), yet dietary ALA remains a significant contributor to tissue EPA/DHA status, particularly in populations with low fish consumption. Beyond serving as membrane components, EPA and DHA are substrates for enzymatic oxygenation by cyclooxygenase (COX), lipoxygenase (LOX), and cytochrome P450 enzymes, giving rise to specialized pro-resolving mediators (SPMs), including resolvins (E-series from EPA, D-series from DHA), protectins, and maresins. These SPMs actively resolve inflammation by promoting macrophage efferocytosis, suppressing neutrophil infiltration, and downregulating NF-κB-dependent pro-inflammatory cytokine production (Serhan, 2015). In the context of cancer, the ALA–EPA–DHA–SPM axis is increasingly recognized as a key mechanistic link between omega-3 fatty acid intake and reduced cancer risk, acting through both direct anti-proliferative effects on tumor cells and indirectly anti-inflammatory remodeling of the TME.

Beyond direct effects on cancer cells, PSO and its omega-3 constituents exert profound immunomodulatory effects on the tumor immune microenvironment (TIME). EPA and DHA are incorporated into the phospholipid membranes of immune cells, where they alter lipid raft organization, downstream receptor signaling, and eicosanoid profiles. Specifically, omega-3 fatty acids competitively inhibit arachidonic acid (AA) metabolism, shifting the prostaglandin and leukotriene balance from pro-inflammatory (PGE_2_, LTB_4_) toward less inflammatory or anti-inflammatory species (PGE_3_, LTB_5_) (Calder, 2017). This eicosanoid class switch has direct implications for cancer immunity: PGE_2_, a well-established immunosuppressive lipid mediator that promotes myeloid-derived suppressor cell (MDSC) accumulation and impairs CD8^+^ T-cell cytotoxicity, is suppressed by EPA/DHA competition at the COX-2 level. Preclinical studies have shown that dietary omega-3 supplementation enhances anti-tumor immunity by increasing tumor infiltration of CD8^+^ T cells and natural killer (NK) cells while reducing regulatory T cell (Treg) and MDSC populations (Fabian et al., 2015). In the context of colitis-associated colorectal cancer, Korsirikoon et al. (Korsirikoon et al., 2024) demonstrated that PSO supplementation significantly increased the abundance of beneficial short-chain fatty acid (SCFA)-producing gut microbiota, including *Lactobacillus* and Bifidobacterium species, while reducing pro-inflammatory taxa such as Desulfovibrio and *Enterococcus*. This microbiota remodeling was correlated with reduced colonic IL-6 and TNF-α levels and diminished tumor burden, suggesting that PSO’s anticancer effects are at least partially mediated through gut–immune axis modulation.

An emerging and clinically relevant aspect of perilla pharmacology is the synergistic interaction between PSO-derived omega-3 fatty acids and other perilla bioactive compounds. Tantipaiboonwong et al. (Tantipaiboonwong et al., 2021) provided direct evidence for this synergy by demonstrating that a combined formulation of cold-pressed PSO and a rosmarinic acid-rich fraction from perilla leaves exhibited significantly greater anti-proliferative and pro-apoptotic activity against A549 lung adenocarcinoma cells compared to either component alone. Mechanistically, RA potentiates ALA’s anti-inflammatory effects by suppressing NF-κB activation at a transcriptional level, thereby amplifying the downstream anti-inflammatory consequences of omega-3 eicosanoid class switching. Conversely, omega-3 fatty acids enhance the bioavailability and cellular uptake of polyphenolic compounds by modulating membrane fluidity and tight junction permeability, potentially addressing the well-documented bioavailability limitation of RA and other perilla polyphenols (Rein et al., 2013). The polyphenol–omega-3 synergy extends to ferroptosis regulation: omega-3 PUFAs, being highly susceptible to lipid peroxidation, can serve as substrates for ferroptotic cell death, and their co-administration with ferroptosis-inducing perilla compounds such as PAH may provide an additional axis for therapeutic augmentation. This multi-level synergy—encompassing complementary mechanisms, enhanced bioavailability, and shared pathway convergence—positions PSO not merely as an inert lipid vehicle but as an active pharmacological partner that amplifies the therapeutic potential of the broader perilla phytochemical arsenal.

Additionally, the synergistic effects of combining PSO with other perilla-derived phytochemicals have been explored. Tantipaiboonwong et al. (Tantipaiboonwong et al., 2021) reported that ALA-rich PSO and rosmarinic acid-rich fractions extracted from perilla seed meal cooperatively suppress TNF-alpha-induced oxidative stress and inflammation in A549 lung adenocarcinoma cells (Tantipaiboonwong et al., 2021), suggesting that the whole-plant extracts may offer superior therapeutic benefits through multi-component synergism.

### 2.5 Summary of perilla bioactive compounds

Table 1 summarizes the major bioactive compounds of *Perilla frutescens* discussed in this section, including their chemical classification, plant source, and key anticancer activities. The major classes of bioactive compounds and their associated biological activities are illustrated in Figure 2.

**TABLE 1:** Major bioactive compounds of Perilla frutescens with anticancer activities.

Compound	Class	Source	Formula	Key anticancer mechanisms	Key cancers
Perillaldehyde (PAH)	Monoterpene	Leaves (50%–70%)	C_10_H_14_O	Ferroptosis (Xc-/GSH/GPX4); autophagy (AMPK, PI3K); apoptosis (HSP70); RANKL	Gastric, CRC, AML, CML, prostate
Rosmarinic acid (RA)	Phenolic acid	Leaves and seeds	C_18_H_16_O_8_	KRAS(G12C) binding; Src inactivation; NF-kB; IL-17A-ferroptosis	NSCLC, TNBC, A549
Luteolin	Flavonoid	Leaves	C_15_H_10_O_6_	PI3K/AKT; NF-kB; p53; MAPK; epigenetic	HCC, breast, various
Apigenin	Flavonoid	Leaves	C_15_H_10_O_5_	PI3K/AKT; JAK-STAT; Hippo; cell cycle arrest	Breast, various
ALA	Omega-3 PUFA	Seed oil (50%–60%)	C_18_H_30_O_2_	NF-kB; gut microbiome; synergy with RA	Colorectal
PLE (extract)	Multi-comp	Leaves (EtOH)	Complex	Hippo-YAP/TAZ (LATS1/2); multi-pathway; synergy	Breast (TNBC)

**FIGURE 2:**
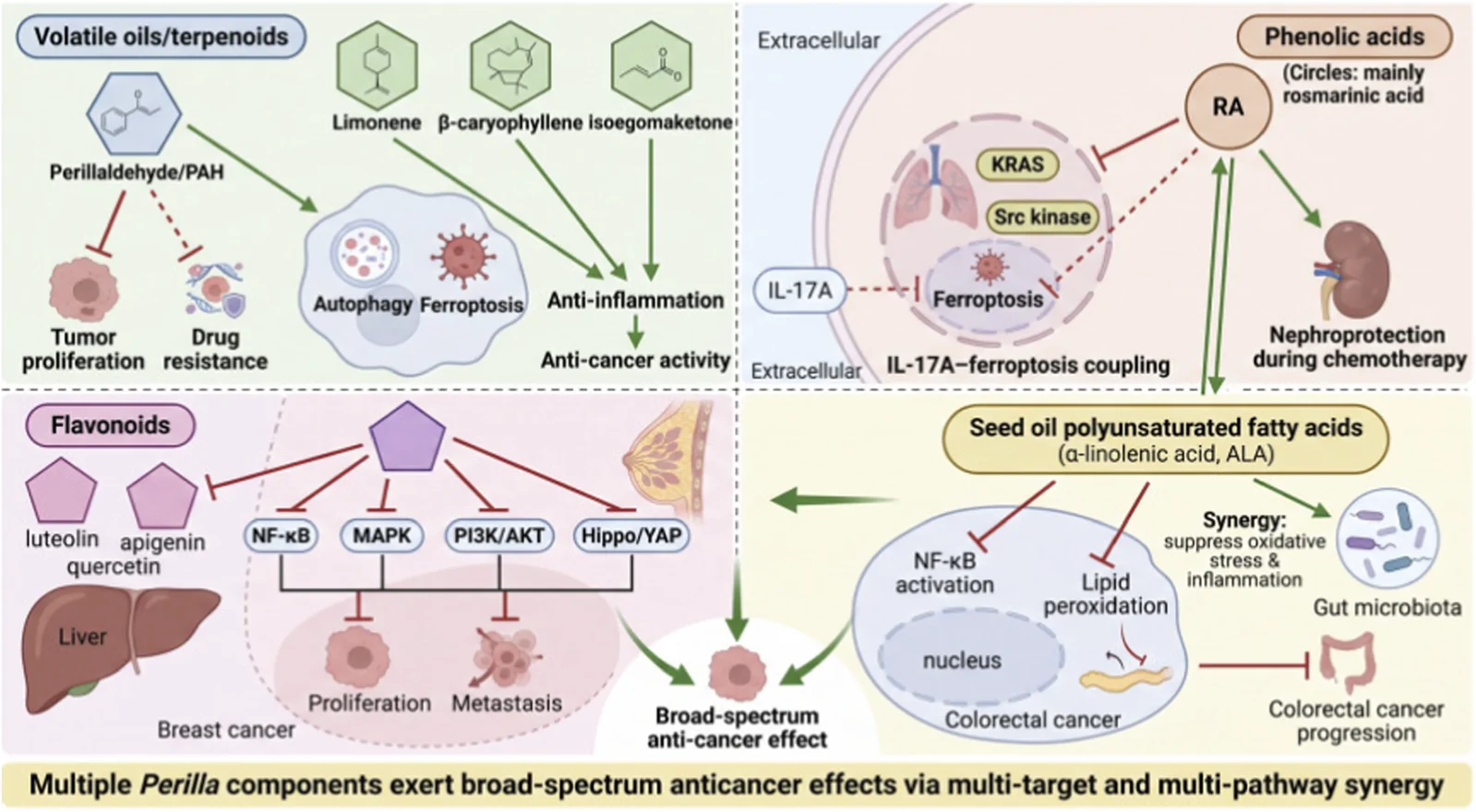
Major bioactive compounds of Perilla frutescens with anticancer activity.

## 3 Regulation of programmed cell death by perilla compounds

Programmed cell death (PCD) is a fundamental biological process that eliminates damaged, unnecessary, or potentially malignant cells, and its dysregulation is a hallmark of cancer. The induction of PCD represents a primary strategy for anticancer therapy, and natural products have emerged as versatile modulators of multiple cell death pathways. Perilla-derived bioactive compounds have been demonstrated to regulate three major forms of PCD—apoptosis, autophagy, and ferroptosis—through distinct yet interconnected molecular mechanisms. This section systematically examines how perilla compounds orchestrate these cell death modalities and explores the emerging concept of cell death crosstalk in cancer therapy.

### 3.1 Apoptosis

Apoptosis (type I PCD) involves cell shrinkage, nuclear condensation, DNA fragmentation, and apoptotic body formation with phagocytic clearance. Two principal pathways initiate apoptosis: the intrinsic (mitochondrial) pathway, governed by Bcl-2 family proteins controlling MOMP, cytochrome c release, and caspase-9 activation; and the extrinsic (death receptor) pathway, mediated by Fas and TRAIL-R recruiting caspase-8 via DISC. Perilla-derived compounds engage both pathways across multiple cancer types (Huang et al., 2026).

#### 3.1.1 Perillaldehyde-induced apoptosis

PAH has been demonstrated to induce apoptosis through multiple mechanisms depending on the cancer cell context. In colorectal cancer (CRC), Leng et al. (Leng et al., 2025) showed that PAH treatment significantly suppressed CRC cell proliferation and colony formation *in vitro* and inhibited xenograft tumor growth *in vivo*. Transcriptomic analysis revealed that PAH modulated the PI3K/AKT signaling pathway, and subsequent investigations confirmed that this effect was mediated through the downregulation of SRD5A1, a steroid 5-alpha-reductase that was identified as a direct molecular target of PAH via molecular docking and cellular thermal shift assay (CETSA). Importantly, PAH-induced PI3K/AKT suppression was linked to the induction of autophagy rather than canonical apoptosis in this context, highlighting the intricate relationship between different cell death pathways.

In prostate cancer, Lin et al. (Lin et al., 2022) reported that PAH inhibited cell viability and induced apoptosis in PC-3 and DU145 cell lines. The pro-apoptotic effect was associated with the suppression of RANKL signaling-induced osteoclastogenesis, suggesting a dual role of PAH in directly targeting cancer cells while simultaneously inhibiting the bone microenvironment that supports metastatic growth. Furthermore, Zhang et al. (Zhang et al., 2019) demonstrated that PAH suppressed gastric cancer cell growth through AMPK activation, which led to autophagy-mediated cell death rather than classical apoptosis, indicating that the dominant cell death modality induced by PAH is highly context-dependent.

#### 3.1.2 Rosmarinic acid-induced apoptosis

RA exhibits potent pro-apoptotic activity across multiple cancers. Zhao et al. (Zhao et al., 2026) showed that RA directly targets KRAS(G12C) using ABPP coupled with click chemistry, disrupting KRAS/AKT/ERK signaling and inducing apoptosis in NSCLC both *in vitro* and *in vivo*. This represents one of the first demonstrations of a natural polyphenol directly targeting an oncogenic mutant protein. In breast cancer, Jeong et al. (Jeong et al., 2026) demonstrated RA inhibits norepinephrine-induced TNBC migration through Src inactivation. RA-rich fractions from perilla seed meal combined with ALA-rich seed oil synergistically suppress TNF-α-induced oxidative stress in A549 cells.

In breast cancer, Jeong et al. (Jeong et al., 2026) demonstrated that RA inhibited norepinephrine-induced migration and invasion in triple-negative breast cancer (TNBC) cells through Src kinase inactivation. Although this study focused primarily on metastasis suppression, the underlying mechanism involved the modulation of survival signaling that can potentiate apoptotic responses under conditions of cellular stress. A study by Tantipaiboonwong et al. (Tantipaiboonwong et al., 2021) further showed that RA-rich fractions from perilla seed meal, when combined with ALA-rich PSO, exhibited synergistic effects in suppressing TNF-alpha-induced oxidative stress and inflammation in A549 lung adenocarcinoma cells, conditions that can sensitize cancer cells to apoptosis.

#### 3.1.3 Flavonoid-induced apoptosis

Perilla flavonoids (luteolin, apigenin) are established apoptosis inducers. Han et al. (Han et al., 2025) used network pharmacology and transcriptome sequencing to identify luteolin targets in HCC, revealing modulation of p53, PI3K/AKT, and MAPK pathways. Kim et al. (Kim et al., 2021) showed that perilla leaf ethanol extract (PLE) induced apoptosis in MDA-MB-231 and BT549 TNBC cells via Hippo-YAP/TAZ signaling. PLE promoted YAP/TAZ phosphorylation in a LATS1/2-dependent manner, suppressing YAP-TEAD transcriptional activity. The apoptosis-related mechanisms discussed above are summarized in Figure 3.

**FIGURE 3:**
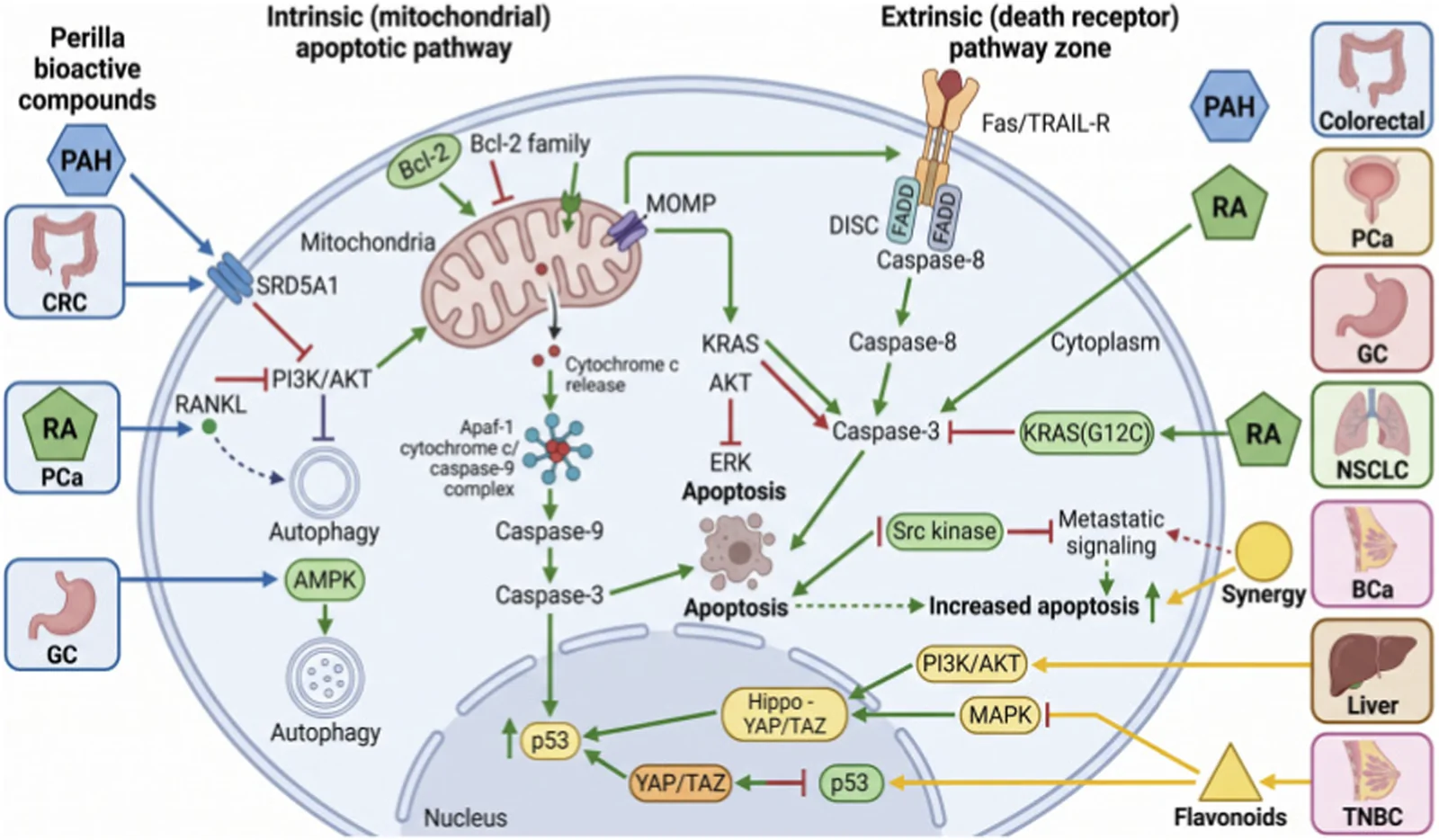
Schematic diagram of the molecular mechanism by which active components from Perilla frutescens induce apoptosis in cancer cells.

#### 3.1.4 Unresolved issues and controversies

Despite substantial evidence, several uncertainties persist. First, PAH appears to engage distinct upstream nodes (AMPK, SRD5A1/PI3K/AKT, RANKL) across different cancer types, yet it remains unclear whether these differences reflect genuine cell-type-specific target selectivity or simply varying experimental conditions. Second, while RA directly targets KRAS(G12C) in NSCLC, whether this represents a unique activity or is shared with other RAS mutants has not been explored. Third, the precise molecular target(s) responsible for perilla flavonoid-induced apoptosis—beyond the Hippo-YAP/TAZ node—remain elusive. Additionally, most mechanistic data derive from short-term monolayer cultures, leaving the impact of the TME and *in vivo* pharmacokinetics largely understudied. Integrating isogenic models and multiomics approaches will be essential to resolve these controversies.

### 3.2 Autophagy

Autophagy delivers cytoplasmic components to lysosomes for degradation. Its role in cancer is dual: tumor-suppressive (eliminating damaged organelles) or pro-survival (under metabolic stress), with significant implications for therapy (Kumar et al., 2025). Perilla-derived compounds, particularly PAH, are important autophagy regulators.

#### 3.2.1 PAH-induced autophagy via AMPK pathway

Zhang et al. (Zhang et al., 2019) showed that PAH suppressed gastric cancer growth by activating AMPK, a central energy sensor that positively regulates autophagy via mTORC1 inhibition. PAH increased AMPK Thr172 phosphorylation, suppressed mTOR signaling, and induced autophagy. Pharmacological inhibition or siRNA knockdown of AMPK attenuated these effects, confirming AMPK as a necessary mediator. Notably, co-treatment with the autophagy inhibitor 3-MA enhanced PAH cytotoxicity, indicating that PAH-induced autophagy is cytoprotective in gastric cancer cells.

#### 3.2.2 PAH-Induced autophagy via SRD5A1/PI3K/AKT pathway

Leng et al. (Leng et al., 2025) identified SRD5A1 as a PAH target in CRC through RNA-seq and bioinformatics. Molecular docking, dynamics simulations, and CETSA confirmed stable PAH-SRD5A1 binding. PAH reduced SRD5A1 expression, suppressing PI3K/AKT signaling and promoting autophagy (autophagosome formation by TEM, LC3-II conversion, p62 degradation). In contrast to gastric cancer, PAH-induced autophagy in CRC was growth-inhibitory; autophagy inhibition partially reversed PAH’s anti-proliferative effects, demonstrating context-dependent autophagy function.

#### 3.2.3 The dual role of autophagy in perilla compound-mediated effects

These contrasting PAH findings illustrate the dual nature of autophagy in cancer. In gastric cancer, PAH-induced autophagy was protective (inhibition enhanced cytotoxicity); in CRC, it contributed to growth inhibition (Figure 4). This dichotomy reflects influences of autophagic flux intensity, cellular metabolic state, p53 status, and specific molecular targets engaged by the compound. The context-dependent duality of autophagy in cancer raises fundamental questions about the underlying determinants of this functional switch.

**FIGURE 4:**
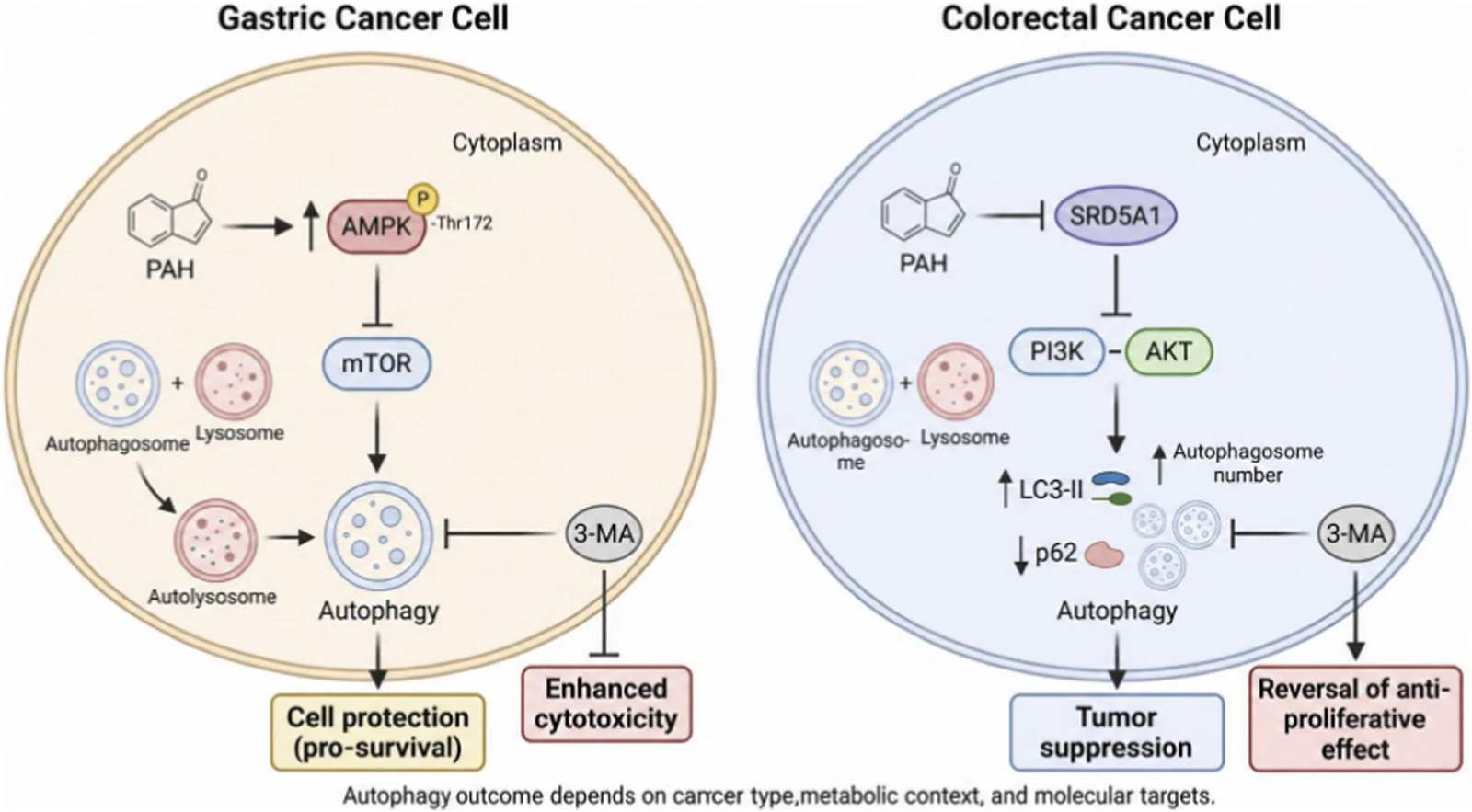
PAH induces autophagy in various types of cancer.

A brief discussion of the confounding factors that may contribute to this discrepant phenomenon is warranted. First, studies on gastric and colorectal cancers employed different cell line panels, drug concentrations, and treatment durations; because PAH is a reactive monoterpene aldehyde, higher concentrations or prolonged exposure may produce off-target effects that alter autophagic outcome. Second, the methods used to assess autophagy differed: LC3-II accumulation and p62 degradation alone cannot distinguish enhanced autophagic flux from blocked autophagosome turnover, and 3-methyladenine, widely used as an autophagy inhibitor, has off-target activities beyond class III PI3K inhibition. Third, basal autophagic activity and p53 status vary across cell lines, and the distinct upstream pathways engaged by PAH—AMPK in gastric cancer *versus* SRD5A1/PI3K/AKT in colorectal cancer—may generate qualitatively different autophagy. Fourth, *in vitro* culture conditions (serum, glucose, and oxygen levels) modulate AMPK and mTORC1 tone and could bias the results. Comparative studies using matched isogenic pairs and standardized autophagic-flux assays are therefore required before the cancer-type-dependent dichotomy can be attributed to intrinsic biological differences rather than experimental variability.

#### 3.2.4 Issues and controversies to be solved

Despite the documented dichotomy, several critical issues remain unresolved. First, it is unclear whether the functional switch of PAH-induced autophagy is dictated primarily by basal autophagic flux, p53 mutational status, metabolic phenotype, or the specific upstream target (AMPK vs. SRD5A1/PI3K/AKT). Second, the interplay among these determinants—for example, how mutant p53 might modulate AMPK or PI3K-dependent autophagy—has not been systematically examined. Third, most data derive from monolayer cultures, leaving the influence of TME heterogeneity (e.g., hypoxia, nutrient gradients) largely unexplored. Addressing these controversies will require comparative isogenic models and dynamic flux measurements to predict when autophagy inhibition is therapeutically beneficial.

### 3.3 Ferroptosis

Ferroptosis is a non-apoptotic regulated cell death (Tang et al., 2021). Dixon, S. J. et al. (Dixon et al., 2012) characterized this process by iron-dependent lipid peroxide accumulation. Its hallmarks include: (i) GSH depletion or GPX4 inactivation; (ii) iron-dependent lipid ROS accumulation; and (iii) polyunsaturated fatty acid-containing phospholipid oxidation (Jiang et al., 2021). Ferroptosis induction is especially promising for apoptosis-resistant cancers. Perilla-derived compounds, particularly PAH, are novel ferroptosis inducers (Catanzaro et al., 2022).

#### 3.3.1 Perillaldehyde as a ferroptosis inducer

Catanzaro et al. (Catanzaro et al., 2022) first identified PAH as a ferroptosis inducer in AML cell lines and primary leukemic cells. PAH increased lipid peroxidation, depleted GSH, and downregulated GPX4. Ferroptosis inhibitor ferrostatin-1 (Fer-1), but not apoptosis (Z-VAD-FMK) or necroptosis (necrostatin-1) inhibitors, blocked PAH-induced death (Catanzaro et al., 2022). PAH synergized with RSL3 (ferroptosis inducer), while iron chelation (deferoxamine) or GPX4 overexpression conferred resistance, establishing PAH as the first monoterpene natural product capable of ferroptosis induction.

Wang et al. (Wang et al., 2025) comprehensively characterized PAH-induced ferroptosis in gastric cancer (AGS, HGC27, MFC). PAH inhibited viability, proliferation, and migration *in vitro* and suppressed subcutaneous xenograft growth in BALB/c nude mice. Mechanistically, PAH reduced MMP, accumulated ROS, depleted GSH, increased lipid peroxidation and Fe2+ levels—consistent with System Xc-/GSH/GPX4 axis inhibition. PAH also regulated ferroptosis through P62-Keap1-Nrf2 signaling, modulating iron transport (TFR1) and storage (FTH1) proteins. Fer-1 partially rescued PAH-induced death, while RSL3 synergistically enhanced cytotoxicity, suggesting ferroptosis-based combination strategies for gastric cancer.

Importantly, Wang et al. (Wang et al., 2025) further elucidated that PAH regulated ferroptosis through the P62-Keap1-Nrf2 signaling pathway. PAH treatment influenced the expression of proteins involved in iron transport (transferrin receptor 1, TFR1) and iron storage (ferritin heavy chain, FTH1), indicating a comprehensive modulation of cellular iron homeostasis. The functional significance of ferroptosis in PAH’s anticancer effects was confirmed by the observation that Fer-1 partially rescued PAH-induced cell death, whereas the ferroptosis inducer RSL3 synergistically enhanced PAH cytotoxicity. These findings suggest that combination strategies involving PAH and ferroptosis-inducing agents could represent a novel therapeutic approach for gastric cancer.

#### 3.3.2 Rosmarinic acid and ferroptosis regulation

RA also engages ferroptosis regulation. Süzen Celbek et al. (Süzen Celbek et al., 2026) showed that RA protects renal cells from ifosfamide-induced ferroptotic damage by disrupting IL-17A-ferroptosis coupling, demonstrating context-dependent ferroptosis modulation—protecting normal cells while potentially inducing ferroptosis in cancer cells—a therapeutic advantage of natural compounds.

#### 3.3.3 Therapeutic implications of ferroptosis induction

Ferroptosis induction by PAH is particularly attractive for apoptosis-resistant cancers (p53 mutant, Bcl-2 overexpressing). Zhou et al. (Zhou et al., 2026) reviewed Chinese herbal medicine components targeting ferroptosis, and Zhang et al. (2026) comprehensively summarized ferroptosis-inducing compounds (Zhang et al., 2026), collectively positioning PAH as a promising ferroptosis-based anticancer lead (Figure 5).

**FIGURE 5:**
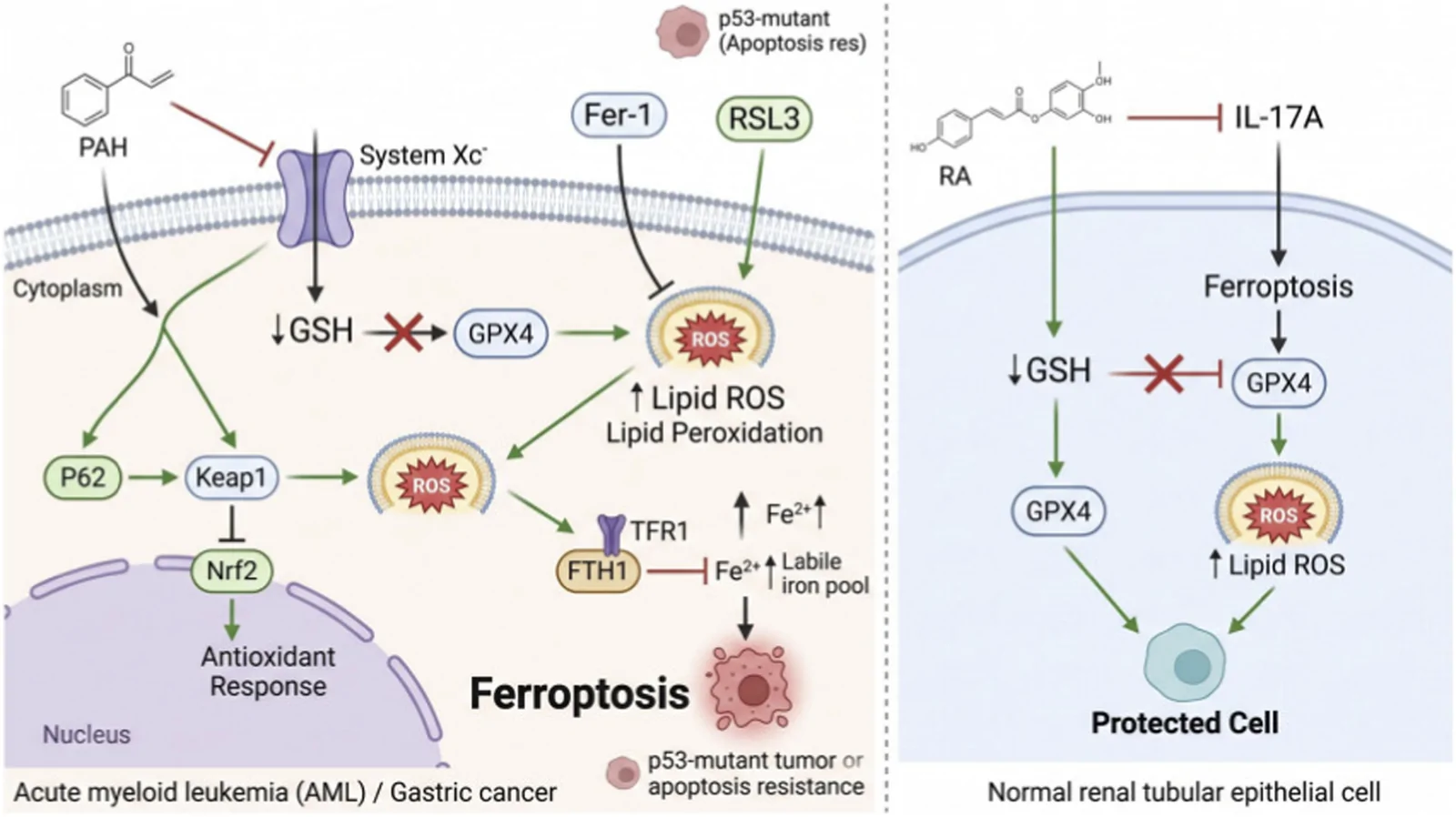
PAH-induced ferroptosis provides a novel strategy for apoptosis-resistant tumors.

#### 3.3.4 Unresolved issues and controversies about PAH-triggered ferroptosis

Despite compelling evidence for PAH as a ferroptosis inducer, several critical questions remain. First, the precise molecular target(s) responsible for PAH-triggered ferroptosis remain to be identified. Current evidence indicates that PAH suppresses the function of the System Xc^−^–GSH–GPX4 axis, possibly through indirect regulation of the P62–Keap1–Nrf2 pathway, while its direct target protein remains to be identified; it is also unclear whether this mechanism is universal across cancer types or cell-line dependent. Second, the reported protective effect of RA against chemotherapy-induced ferroptosis in normal renal cells, raises a paradoxical possibility: could RA exert opposite effects on ferroptosis in malignant *versus* non-malignant cells, and if so, what determines this selectivity? Third, the interplay between ferroptosis and other PAH-induced death modalities (apoptosis, autophagy) remains poorly understood—are these pathways parallel, sequential, or mutually exclusive, and does their crosstalk influence therapeutic outcomes? Fourth, most data derive from short-term *in vitro* models; the pharmacokinetic profile of PAH, its bioavailability in tumor tissues, and the impact of the TME (e.g., hypoxia, stromal cells) on ferroptosis sensitivity are largely unexplored. Finally, robust biomarkers to predict ferroptosis responsiveness in patients are lacking, which complicates the translation of PAH-based ferroptosis strategies into clinical practice. Addressing these controversies will require integrative studies combining isogenic cell systems, *in vivo* imaging of lipid peroxidation, and patient-derived models.

### 3.4 Crosstalk among apoptosis, autophagy, and ferroptosis

A distinctive feature of perilla compounds is their simultaneous engagement of multiple cell death pathways (Qiao et al., 2026). Understanding apoptosis-autophagy-ferroptosis crosstalk is essential for optimizing therapeutic strategies. Autophagy contributes to ferroptosis through ferritinophagy (releasing free iron, enhancing lipid peroxidation), while ferroptosis can trigger autophagy as a stress response. The P62-Keap1-Nrf2 pathway—modulated by PAH—connects oxidative stress, autophagy, and ferroptosis. P62 (SQSTM1), when accumulated due to impaired autophagy, sequesters Keap1, activating Nrf2 and an antioxidant program that counteracts ferroptosis.

The interplay between autophagy and ferroptosis is particularly complex. Autophagy can contribute to ferroptosis through selective degradation of ferritin (a process termed ferritinophagy), which releases free iron and enhances lipid peroxidation. Several studies have shown that autophagy induction can sensitize cancer cells to ferroptosis by increasing the labile iron pool. Conversely, ferroptosis can also trigger autophagy as a stress response. The P62-Keap1-Nrf2 pathway, which was shown to be modulated by PAH in gastric cancer, serves as a critical node connecting oxidative stress responses, autophagy, and ferroptosis. P62 (also known as SQSTM1) is an autophagy receptor that targets ubiquitinated proteins for autophagic degradation (Kumar et al., 2022), but when accumulated due to impaired autophagy, it can sequester Keap1, leading to Nrf2 activation and an antioxidant transcriptional program that counteracts ferroptosis (Jiang et al., 2024).

The PI3K/AKT/mTOR pathway governs the apoptosis-autophagy-ferroptosis balance. PAH-induced PI3K/AKT suppression promoted autophagy in CRC, while AKT signaling modulates ferroptosis sensitivity through GSH metabolism and GPX4 expression. AMPK activation by PAH serves as a metabolic checkpoint promoting autophagy (via mTORC1 inhibition) and influencing ferroptosis through lipid metabolism.

The dual role of autophagy—either cytoprotective or cytotoxic—is a critical consideration. In gastric cancer, PAH-induced autophagy was protective, and its inhibition enhanced cell death, suggesting that combining PAH with autophagy inhibitors could improve therapeutic efficacy. In contrast, in CRC, autophagy contributed to PAH’s growth-inhibitory effects, indicating that autophagy induction itself is a therapeutic mechanism. For ferroptosis, the combination of PAH with ferroptosis inducers such as RSL3 synergistically enhanced cell death, suggesting that ferroptosis-based combination strategies may be particularly effective. These observations underscore the importance of cancer type-specific considerations when developing therapeutic strategies involving perilla compounds.

Several biological determinants are likely to shape the functional switch of PAH-mediated autophagy and merit systematic investigation. p53 status: wild-type p53 promotes autophagy through AMPK activation and transcriptional targets such as DRAM and Sestrin2, whereas mutant p53 can impair or redirect autophagic responses; the gastric and colorectal cancer cell lines used in PAH studies differ in TP53 status, complicating cross-study interpretation. Cellular metabolic background: glycolytic and oxidative cancer cells differ in AMP/ATP ratios and basal AMPK–mTORC1 tone, which may determine whether PAH-induced AMPK activation translates into cytoprotective or cytotoxic autophagy. Drug concentration and exposure time: low-dose, short-term PAH may elicit homeostatic (cytoprotective) autophagy, whereas higher concentrations or prolonged exposure may saturate lysosomal capacity and shift the balance toward autophagic cell death; systematic dose–response and time-course data are largely lacking. TME: hypoxia and nutrient deprivation, hallmarks of solid tumors, constitutively activate autophagy as a survival program, and stromal and immune cells may further modify cancer-cell autophagic responses; almost all available data derive from normoxic monolayer cultures. Key limitations of the current evidence should be explicitly acknowledged, including the absence of autophagic-flux measurements *in vivo*, single time-point analyses, reliance on pharmacological inhibitors with off-target effects, and the lack of isogenic cell models. Addressing these gaps is essential to predict when autophagy inhibition (gastric cancer) or autophagy induction (colorectal cancer) is therapeutically appropriate. Figure 6 summarizes the proposed crosstalk among these cell death pathways, with emphasis on the context-dependent roles of autophagy and ferroptosis.

**FIGURE 6:**
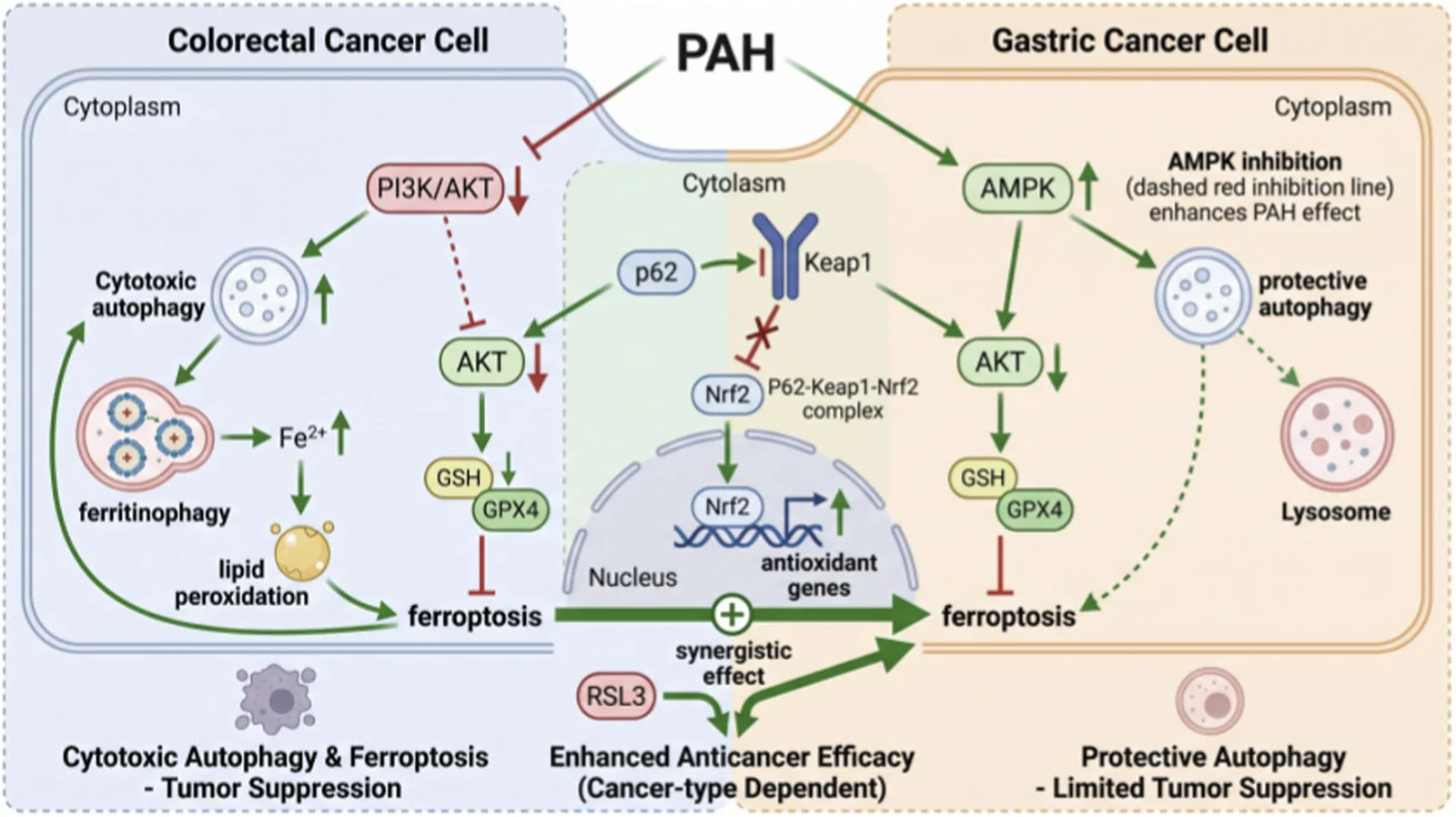
Crosstalk among apoptosis, autophagy, and ferroptosis.

In summary, perilla-derived compounds, particularly PAH and RA, engage a sophisticated network of cell death pathways that extends beyond classical apoptosis to include autophagy and ferroptosis. The ability of these compounds to induce multiple forms of cell death simultaneously makes them particularly attractive for targeting apoptosis-resistant cancers. However, the context-dependent nature of these effects necessitates careful consideration of the specific cancer type, genetic background, and TME when designing therapeutic strategies. The synergistic potential of combining perilla compounds with established cell death modulators (e.g., ferroptosis inducers, autophagy inhibitors) represents a promising direction for future research and clinical translation.

Unresolved Questions and Controversies: Although PAH induces ferroptosis, the relative contribution of this pathway vs. apoptosis in different genetic backgrounds remains unclear, as most studies rely on single-cell lines without isogenic pairs.

## 4 Modulation of key signaling pathways

The anticancer effects of *Perilla frutescens*-derived compounds are mediated through the coordinated modulation of multiple intracellular signaling pathways. Rather than acting through a single linear mechanism, these compounds engage a network of interconnected signal transduction cascades that regulate cell proliferation, survival, metabolism, and death. This systems-level modulation is particularly advantageous for cancer therapy, as it reduces the likelihood of resistance development through compensatory pathway activation. This section examines the major signaling pathways targeted by perilla compounds, with an emphasis on the PI3K/AKT/mTOR, AMPK, Hippo-YAP/TAZ, MAPK/ERK, and NF-kappaB/JAK-STAT networks.

### 4.1 PI3K/AKT/mTOR signaling pathway

The PI3K/AKT/mTOR cascade integrates growth factor, nutrient, and energy signals to coordinate cell growth, proliferation, survival, and metabolism (Fruman et al., 2017). Multiple perilla compounds converge on the PI3K/AKT/mTOR axis. In CRC, PAH directly targets SRD5A1, suppressing AKT/mTORC1 activity; PI3K activation reversed PAH-induced autophagy, confirming pathway specificity. RA exerts dual effects: it binds KRAS(G12C) to inhibit GTP loading and AKT/ERK signaling in NSCLC (Zhao et al., 2026), and inactivates Src to suppress norepinephrine-driven TNBC metastasis. Flavonoids (luteolin, apigenin) reinforce PI3K/AKT inhibition while modulating NF-κB and MAPK to restore redox balance (Binmahfouz, 2026), and suppress the IL-6/JAK/STAT3 axis that drives chemoresistance in aggressive breast cancer (Kubatka et al., 2026). Thus, perilla compounds orchestrate a multi-node blockade of PI3K/AKT and its intersecting pathways (KRAS, Src, JAK-STAT), yielding enhanced efficacy and reduced resistance potential—a network-level rationale for combining them with existing therapies across diverse cancers (Figure 7).

**FIGURE 7:**
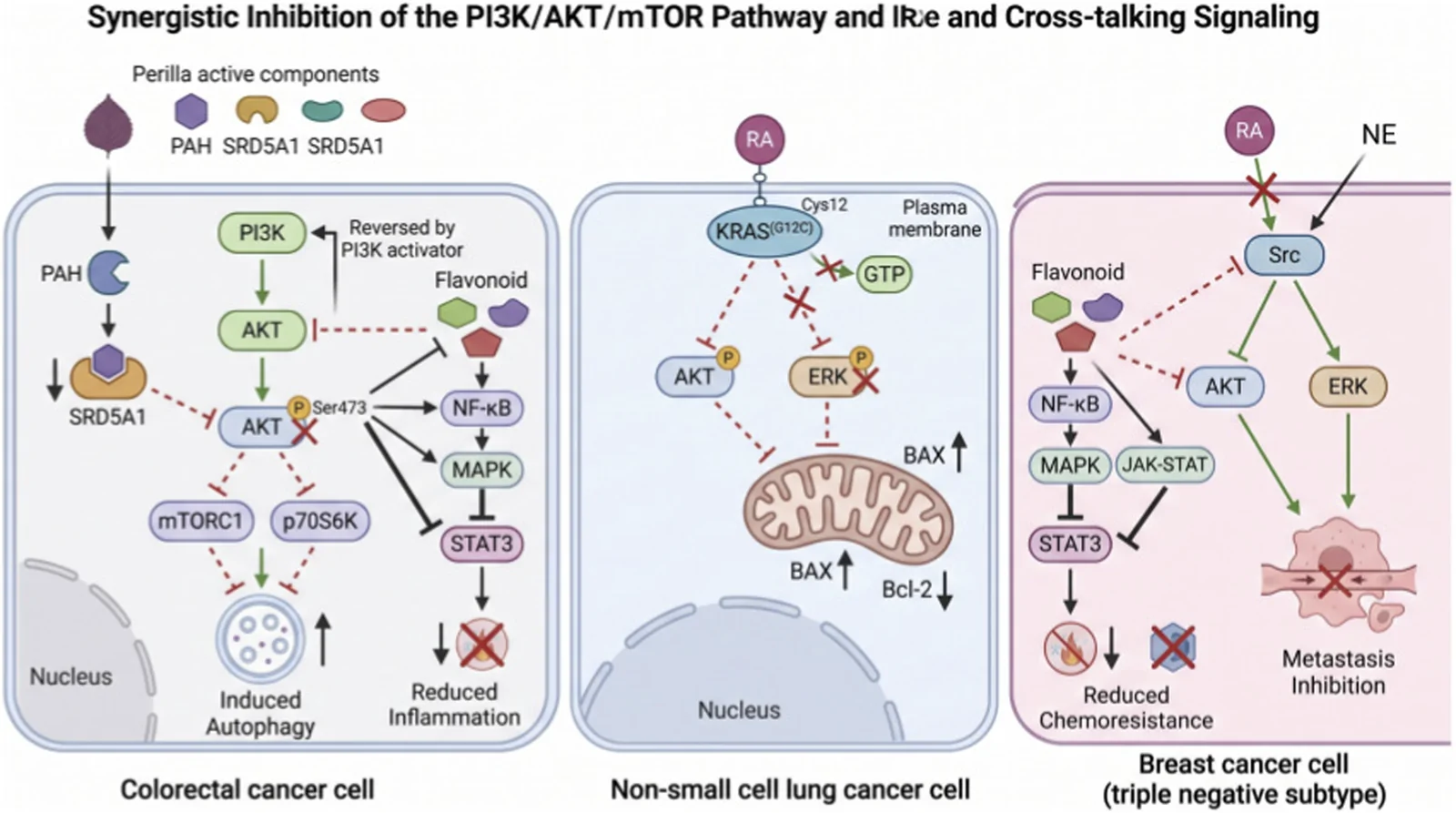
The regulatory effects of active components in perilla (PAH, RA, and flavonoids) on various nodes of the PI3K/AKT/mTOR signaling pathway.

### 4.2 AMP-activated protein kinase (AMPK) signaling

AMP-activated protein kinase (AMPK) is a master regulator of cellular energy homeostasis that is activated under conditions of energy stress (elevated AMP/ATP ratio). Beyond its canonical role in metabolic regulation, AMPK functions as a tumor suppressor by inhibiting the mTOR pathway, promoting autophagy, and regulating cell growth and proliferation. The activation of AMPK by natural compounds has emerged as a promising anticancer strategy.

Zhang et al. (Zhang et al., 2019) demonstrated that PAH acts as a potent AMPK activator in gastric cancer. PAH dose-dependently increased AMPK Thr172 phosphorylation, inhibiting mTORC1 (reduced p70S6K and 4E-BP1 phosphorylation) and inducing autophagy (LC3-II conversion, autophagosome formation). AMPK inhibitor compound C and AMPK-alpha siRNA both attenuated PAH-induced autophagy, confirming pathway specificity (Figure 8). The AMPK-mTOR axis serves as a critical signaling hub intersecting with the ferroptosis network through lipid metabolism and oxidative stress regulation.

**FIGURE 8:**
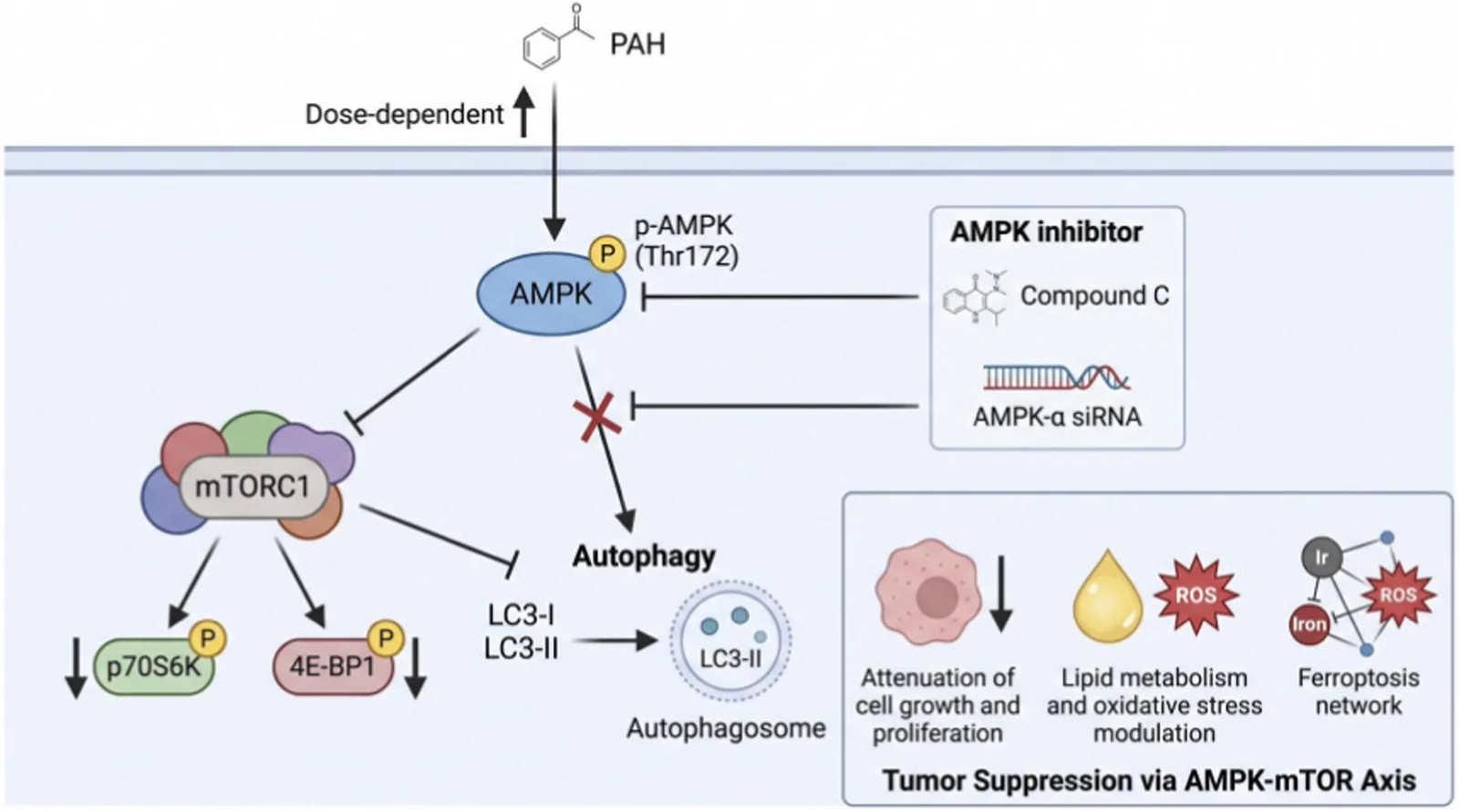
PAH activates AMPK and inhibits the mTOR pathway, thereby exerting an antitumor effect.

### 4.3 Hippo-YAP/TAZ signaling pathway

The Hippo pathway controls organ size and cell proliferation via YAP/TAZ transcriptional co-activators. Active Hippo signaling (MST1/2-LATS1/2 kinase cassette) phosphorylates YAP/TAZ, promoting cytoplasmic retention and degradation. Inactivated Hippo allows YAP/TAZ nuclear translocation, TEAD binding, and target gene expression (CTGF, CYR61, survivin). YAP/TAZ hyperactivation is common in cancers and associated with poor prognosis (Papanikolaou et al., 2026).

Kim et al. (Kim et al., 2021) first demonstrated that perilla leaf extract (PLE) exerts antitumor effects through the Hippo-YAP/TAZ pathway in breast cancer. PLE induced YAP Ser127 and TAZ Ser89 phosphorylation (LATS1/2 substrates), promoting cytoplasmic retention and inactivation, leading to dissociation of YAP/TAZ-TEAD complexes and suppression of target genes (CTGF, CYR61). LATS1/2 double-knockout confirmed pathway specificity. PLE reduced viability of MDA-MB-231 and BT549 cells dose-dependently, induced apoptosis, and inhibited migration—effects significantly weakened in YAP- and TEAD-overexpressing clones, confirming YAP/TAZ as key mediators (Figure 9).

**FIGURE 9:**
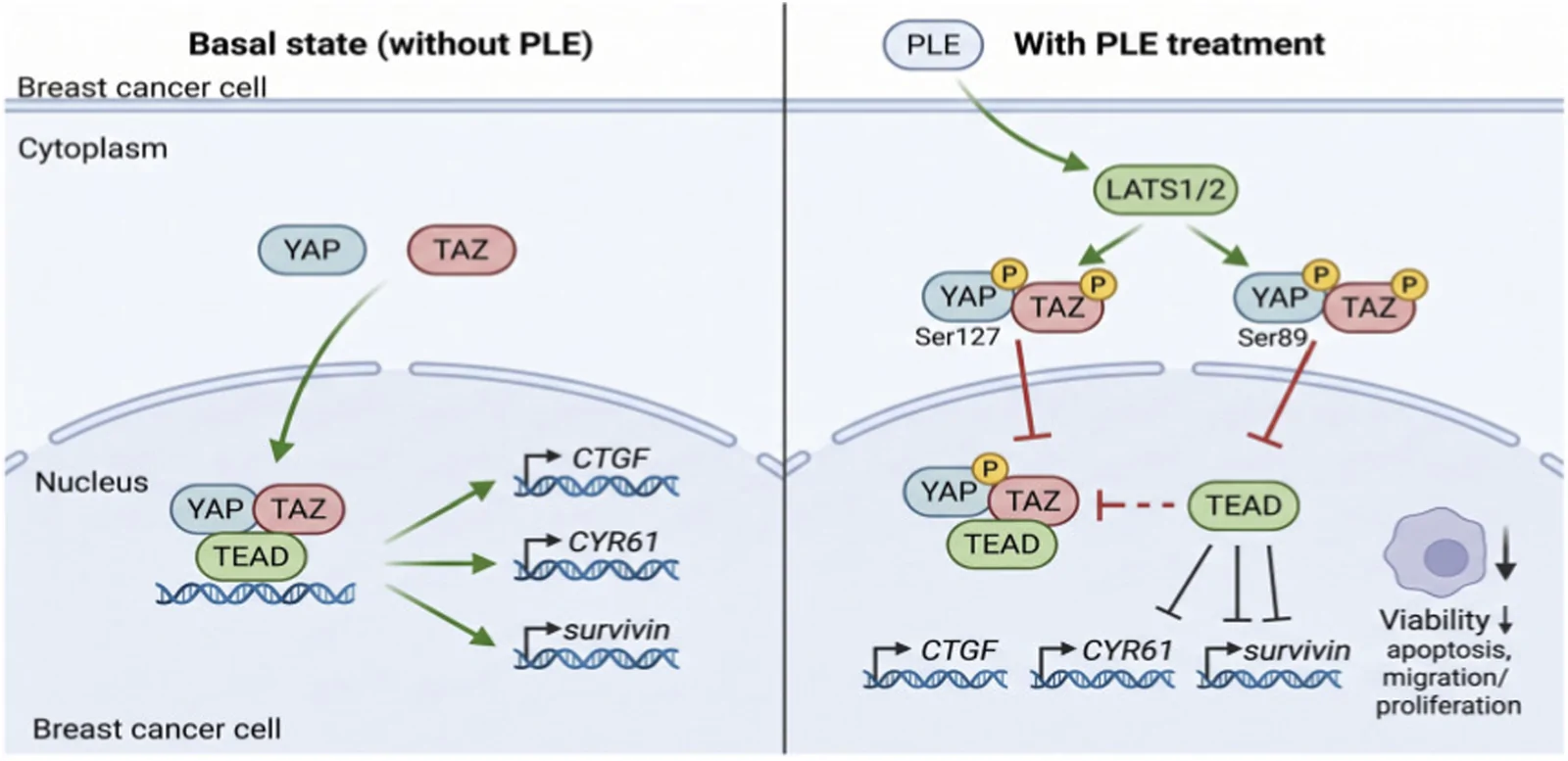
Perilla leaf extract (PLE) exerts antitumor effects in breast cancer through the Hippo-YAP/TAZ pathway.

### 4.4 MAPK/ERK signaling pathway

The three major MAPK pathways—ERK1/2 (proliferation/survival), JNK, and p38 (stress/apoptosis)—play distinct roles in cancer. Perilla compounds modulate MAPK signaling through multiple mechanisms.

RA’s direct targeting of KRAS(G12C) suppresses both AKT and ERK signaling, inhibiting NSCLC proliferation and inducing apoptosis. Luteolin modulates MAPK context-dependently: suppressing ERK1/2 while activating p38 and JNK to promote apoptosis. The flavonoid-rich perilla extract likely contributes to coordinated MAPK modulation.

Luteolin has been shown to modulate MAPK signaling in a context-dependent manner. Binmahfouz (Binmahfouz, 2026) reported that luteolin regulates the NF-kappaB/MAPK signaling axis, exerting dual effects on the inflammatory and oxidative stress responses that are intricately linked to cancer development. Luteolin can suppress ERK1/2 phosphorylation in cancer cells while simultaneously activating p38 and JNK to promote apoptosis, a differential modulation that reflects its multi-target nature. In perilla leaf extract studies, the flavonoid-rich composition likely contributes to the overall modulation of MAPK signaling, complementing the effects of other bioactive constituents (Figure 10).

**FIGURE 10:**
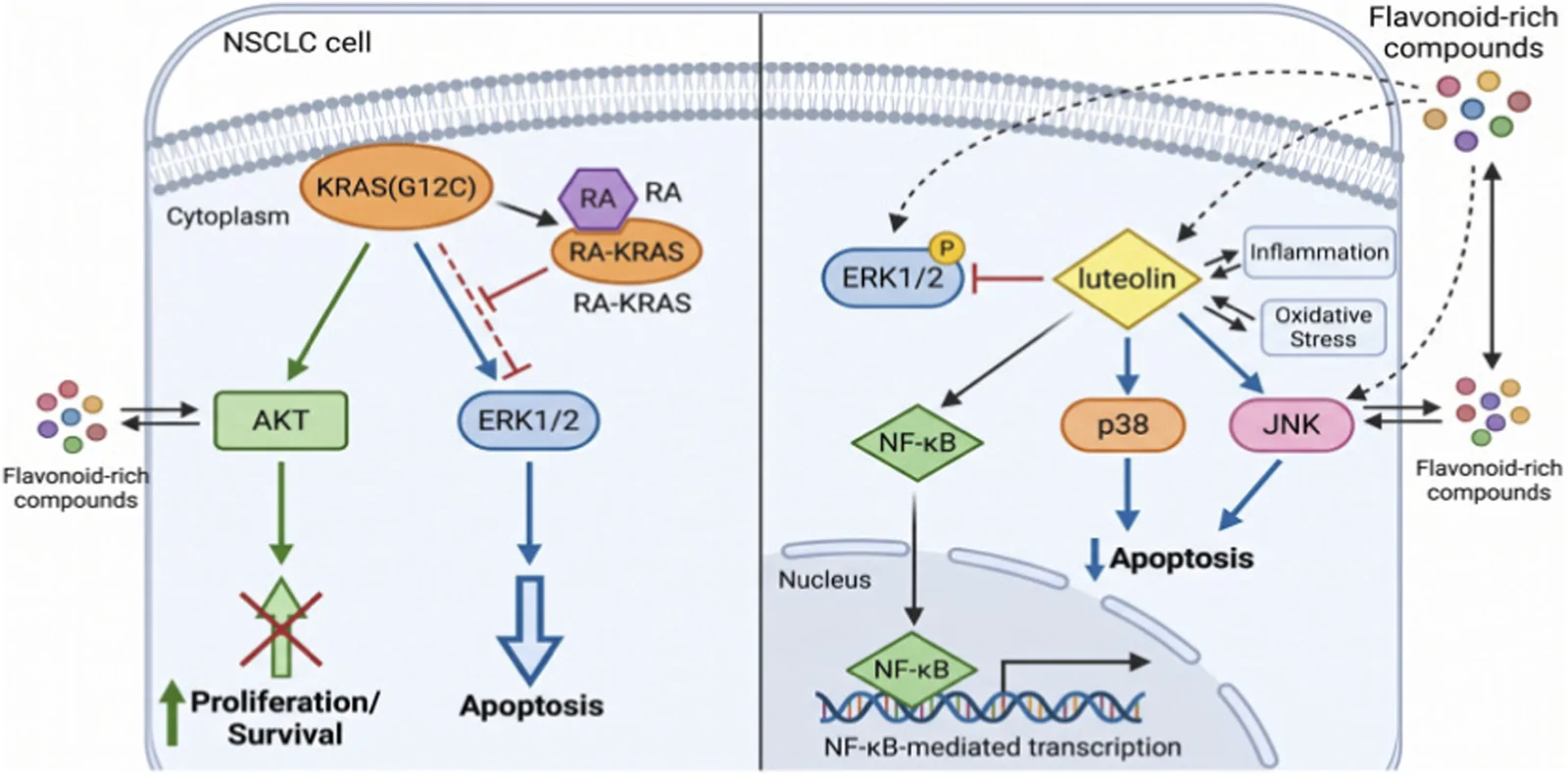
Perilla compounds regulate the three major MAPK signaling pathways through multiple mechanisms.

### 4.5 NF-kappaB and JAK-STAT signaling pathways

NF-κB and JAK-STAT pathways are central mediators of inflammation-driven carcinogenesis. Their crosstalk with oncogenic pathways provides mechanistic basis for chemopreventive effects of anti-inflammatory natural products.

#### 4.5.1 Suppression of NF-kappaB signaling

Thomas et al. (Thomas et al., 2020) showed that perilla oil suppresses NF-κB p65 phosphorylation and nuclear translocation, reducing TNF-α, IL-1β, IL-6, COX-2, and iNOS expression in the colon. RA also inhibits NF-κB by preventing IκBα degradation. Luteolin modulates NF-κB through IKK inhibition and upstream TLR/RIPK suppression (Binmahfouz, 2026). The convergence of multiple perilla compounds on NF-κB suggests enhanced anti-inflammatory effects through multi-component synergism.

The NF-kappaB-modulating effects of perilla compounds are further supported by studies on individual constituents. RA has been shown to inhibit NF-kappaB activation through the prevention of IkappaB-alpha degradation and subsequent p65 nuclear translocation. Luteolin modulates the NF-kappaB pathway through both direct inhibition of IkappaB kinase (IKK) activity and suppression of upstream activators including Toll-like receptors (TLRs) and receptor-interacting protein kinases (RIPKs). The convergence of multiple perilla compounds on NF-kappaB signaling suggests that the whole-plant extracts may exert enhanced anti-inflammatory effects through multi-component synergism.

#### 4.5.2 Modulation of JAK-STAT signaling

Kubatka et al. (Kubatka et al., 2026) demonstrated that perilla-relevant flavonoids (luteolin, apigenin) suppress the IL-6/gp130/JAK/STAT3 axis, reducing STAT3 Tyr705 phosphorylation and downstream cyclin D1, survivin, Bcl-xL, and VEGF expression—inhibiting proliferation, promoting apoptosis, and suppressing angiogenesis. The intersection of JAK-STAT with NF-κB via shared upstream activators (IL-6, TNF-α) and target genes creates an interconnected inflammatory signaling network exploitable through multi-target perilla compounds (Figure 11).

**FIGURE 11:**
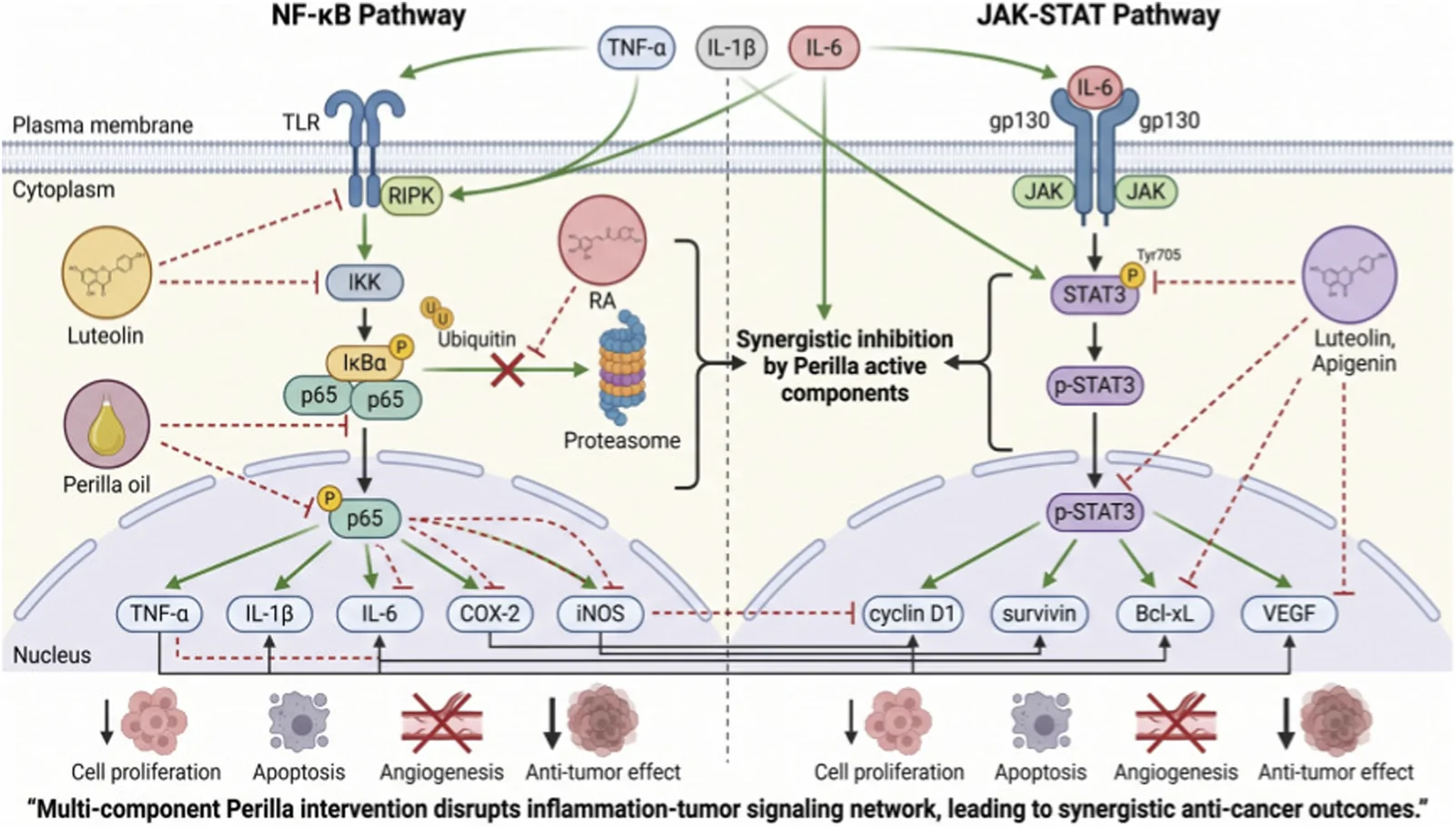
The active components of perilla exert antitumor effects by synergistically inhibiting two major inflammation-related signaling pathways: NF-κB and JAK-STAT.

### 4.6 Summary of signaling pathway modulation

Table 2 provides a consolidated overview of the major signaling pathways modulated by Perilla frutescens-derived compounds, their molecular targets, affected cancer types, and the resulting cellular outcomes.

**TABLE 2:** Signaling pathways modulated by Perilla frutescens-derived compounds.

Pathway	Compound	Target	Effect	Cancer	Outcome	References
PI3K/AKT/mTOR	PAH	SRD5A1	AKT down	CRC	Autophagy	Leng et al., 2025
PI3K/AKT/mTOR	RA	KRAS(G12C)	AKT down	NSCLC	Apoptosis	Zhao et al., 2026
PI3K/AKT/mTOR	RA	Src	AKT/ERK down	TNBC	Migration inhibited	Zhang et al., 2019
PI3K/AKT/mTOR	Luteolin	Multiple	PI3K/AKT down	HCC	Apoptosis	Suzen Celbek et al., 2026
AMPK	PAH	AMPKa	AMPK up, mTOR down	Gastric	Autophagy	Zhang et al., 2025
Hippo-YAP/TAZ	PLE	LATS1/2	YAP/TAZ phos	Breast	Apoptosis	Kim et al., 2021
MAPK/ERK	RA	KRAS(G12C)	ERK down	NSCLC	Proliferation suppressed	Zhao et al., 2026
MAPK/ERK	Luteolin	Multiple	p38/JNK up	Various	Apoptosis	Leong et al., 2026
NF-kB	Perilla oil	p65	Nuc. Transloc. Down	Colon	Inflammation suppressed	Binmahfouz, 2026
NF-kB	RA	IKK	p65, IL-6 down	Multiple	Anti-inflammatory effect	Choi et al., 2025
JAK-STAT	Lut./Apig	STAT3	STAT3 Y705 down	Breast	Apoptosis induced	Jeong et al., 2026

The multi-pathway targeting capacity distinguishes perilla compounds from single-target agents. By simultaneously modulating PI3K/AKT, AMPK, Hippo-YAP/TAZ, MAPK, NF-κB, and JAK-STAT, these compounds create network-level perturbations resilient to compensatory adaptations. The interconnectivity—crosstalk between PI3K/AKT and AMPK via TSC1/2, convergence of Hippo and PI3K/AKT on survival, and integration of NF-κB and JAK-STAT in inflammation—suggests therapeutic effects arise from coordinated network modulation, providing a rational basis for perilla-derived multi-target agents.

## 5 Cancer type-specific effects of perilla-derived compounds

While the previous sections examined the molecular mechanisms and signaling pathways modulated by *Perilla frutescens*-derived compounds in a generalized manner, the antitumor effects of these compounds exhibit considerable cancer type specificity. This selectivity arises from differences in genetic background, tissue-specific signaling dependencies, and the unique TME of each cancer type. Understanding these cancer-specific effects is crucial for the rational design of therapeutic strategies and the identification of patient populations most likely to benefit from perilla-derived therapies. Figure 12 provides an overview of the cancer type-specific effects and associated mechanisms of perilla-derived compounds.

**FIGURE 12:**
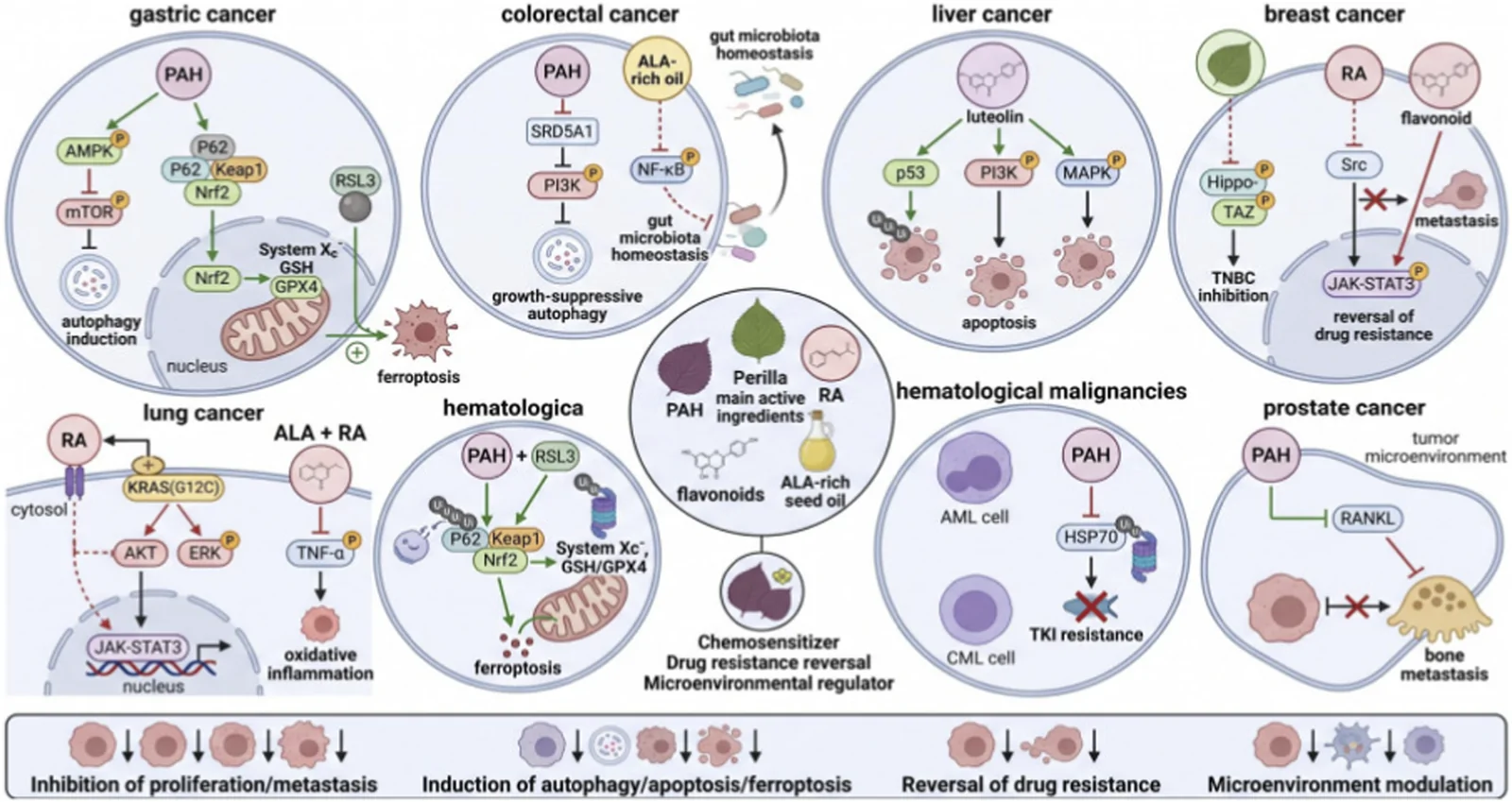
The active components of Perilla frutescens exert broad-spectrum anticancer effects against various malignant tumors through multi-target and multi-mechanism mechanisms.

### 5.1 Digestive system cancers

#### 5.1.1 Gastric cancer

Gastric cancer represents one of the most extensively studied cancer types with respect to perilla compound action (Smyth et al., 2020). Zhang et al. (Zhang et al., 2019) first demonstrated that PAH suppresses gastric cancer cell growth through the AMPK-mTOR-autophagy axis, providing the mechanistic foundation for PAH’s anti-gastric cancer effects. The protective autophagy induced by PAH in this context suggested that combination strategies with autophagy inhibitors could enhance therapeutic efficacy.

Wang et al. (Wang et al., 2025) characterized PAH-induced ferroptosis in gastric cancer (AGS, HGC27, MFC). PAH inhibited growth *in vitro* and *in vivo* via System Xc-/GSH/GPX4 suppression, leading to lipid peroxide and Fe2+ accumulation (Wang et al., 2025). PAH regulated ferroptosis through P62-Keap1-Nrf2 and modulated TFR1 and FTH1 expression. PAH synergized with RSL3, suggesting ferroptosis-based combination strategies may be particularly effective for gastric cancer, which frequently resists conventional therapy.

#### 5.1.2 Colorectal cancer

Colorectal cancer (CRC) has been investigated at multiple levels—from the molecular targeting of cancer cells to the modulation of the TME (Dekker et al., 2019). Leng et al. (Leng et al., 2025) provided mechanistic evidence that PAH inhibits CRC by targeting SRD5A1, leading to PI3K/AKT pathway suppression and the induction of autophagy. Transcriptomic analysis, molecular docking, and cellular thermal shift assays (CETSA) converged to identify SRD5A1 as a direct PAH target, and bioinformatics analysis confirmed the clinical relevance of SRD5A1 expression in CRC prognosis (Leng et al., 2025). The autophagy induced by PAH in CRC was growth-inhibitory, contrasting with the protective autophagy observed in gastric cancer, underscoring the context-dependent nature of PAH’s effects.

Korsirikoon et al. (Korsirikoon et al., 2024) showed that cold-pressed PSO (ALA-enriched) significantly reduced tumor multiplicity and size in AOM/DSS colitis-associated CRC, accompanied by gut microbial homeostasis restoration (increased *Lactobacillus*, Bifidobacterium; decreased pro-inflammatory taxa). Thomas et al. (Thomas et al., 2020) further demonstrated that perilla oil alleviates high-fat diet-induced colonic inflammation through NF-κB suppression, providing mechanistic basis for CRC chemoprevention.

The chemopreventive efficacy of PSO in the AOM/DSS model extended beyond tumor multiplicity reduction. Korsirikoon et al. (Korsirikoon et al., 2024) reported that PSO treatment significantly reduced the incidence of high-grade dysplasia and adenocarcinoma, shifting the histopathological spectrum toward low-grade adenomas. Mechanistically, PSO suppressed NF-κB p65 nuclear translocation in the colonic epithelium, leading to reduced expression of COX-2, iNOS, and pro-inflammatory cytokines (IL-6, TNF-α, IL-1β). Concurrently, PSO restored the expression of the tumor suppressor E-cadherin and reduced nuclear β-catenin localization, indicating suppression of the Wnt/β-catenin pathway—a central driver of colorectal carcinogenesis. These dual anti-inflammatory and Wnt-suppressive effects are consistent with the proposed mechanism of ALA-derived SPMs in resolving chronic colitis-associated inflammation.

The relevance of PSO in CRC extends beyond chemically induced models. In human CRC cell lines (HT-29, Caco-2), ALA and EPA treatment dose-dependently inhibited cell proliferation and induced apoptosis via PPARγ activation and subsequent PTEN upregulation, leading to AKT/mTOR pathway suppression (Calder, 2017). Notably, the combination of ALA with 5-fluorouracil (5-FU), a standard-of-care chemotherapeutic for CRC, produced synergistic cytotoxicity in 5-FU-resistant HCT-116 cells by enhancing oxidative stress and restoring ceramide-mediated apoptosis signaling. This chemosensitizing effect is particularly significant given that resistance to 5-FU remains a major clinical hurdle in CRC management. Furthermore, emerging evidence suggests that the omega-3-mediated remodeling of gut microbiota—increasing butyrate-producing species while reducing pathobionts—may create a colonic microenvironment that is intrinsically less permissive to adenoma–carcinoma progression, positioning PSO as a promising dietary chemopreventive agent for CRC, particularly in individuals with inflammatory bowel disease who are at elevated colorectal cancer risk.

PSO exerts multifaceted immunomodulatory effects on the tumor immune microenvironment (TIME). This eicosanoid class switch has profound consequences for anti-tumor immunity: PGE_2_ promotes myeloid-derived suppressor cell (MDSC) accumulation, M2 macrophage polarization, and CD8^+^ T-cell dysfunction within the TIME. n-3 PUFA supplementation has been shown to reduce pro-tumorigenic immune markers including CD206 and F4/80 in mammary tumor models (Ashraf et al., 2026), and dietary omega-3 PUFAs suppress cholesterol-driven NSCLC progression while enhancing anti-tumor immune responses (Harre et al., 2025). In gastric cancer, bioinformatic and experimental analyses revealed that PUFA-related gene signatures correlate with immune cell infiltration and immunotherapy response (Wu et al., 2025). Furthermore, omega-3 fatty acids released by FAK inhibition in ovarian cancer reprogram resident peritoneal macrophages toward a CXCL13-producing, anti-tumor phenotype (Chen et al., 2026). PSO supplementation suppresses NF-κB-driven pro-inflammatory signaling, reduces COX-2 and iNOS expression, and restores gut microbial homeostasis by enriching SCFA-producing taxa while depleting pro-inflammatory pathobionts (Korsirikoon et al., 2024).

#### 5.1.3 Hepatocellular carcinoma

Han et al. (Han et al., 2025) employed network pharmacology, RNA-seq, and *in vitro* experiments to identify luteolin targets in HCC, revealing p53, PI3K/AKT, and MAPK pathway modulation. *In vitro* validation confirmed luteolin inhibited HCC proliferation and induced apoptosis. Earlier studies also reported perilla leaf extract inhibits HCC via PI3K/AKT suppression (Lin et al., 2007). Apigenin and chrysin in combination attenuate diethylnitrosamine-induced hepatocellular carcinoma through complementary anti-fibrotic and anti-inflammatory mechanisms (Tedik et al., 2026).

### 5.2 Breast cancer

Breast cancer research has been a major focus for perilla-derived compounds, with studies spanning multiple molecular subtypes (Waks and Winer, 2019). Kim et al. (Kim et al., 2021) demonstrated that the ethanol extract of Perilla frutescens leaves (PLE) exerts antitumor effects on triple-negative breast cancer (TNBC) cell lines MDA-MB-231 and BT549 through the Hippo-YAP/TAZ signaling pathway. PLE induced YAP/TAZ phosphorylation in a LATS1/2-dependent manner, suppressed YAP-TEAD transcriptional activity, and inhibited cell migration. Notably, the anti-migratory effect of PLE was significantly attenuated in YAP- and TEAD-overexpressing clones, confirming the pathway specificity of this effect. The study is significant because TNBC lacks targeted therapeutic options and is associated with poor prognosis, making the identification of YAP/TAZ as a druggable vulnerability highly relevant.

Jeong et al. (Jeong et al., 2026) showed that RA inhibits norepinephrine-induced TNBC metastasis through Src kinase inactivation, disrupting the stress-metastasis axis. Perilla flavonoids also modulate JAK-STAT3 signaling, suppressing cancer stem cell properties and reversing chemotherapy resistance, supporting perilla-derived compounds for aggressive breast cancer subtypes with limited treatment options. Importantly, omega-3 PUFAs themselves function as chemosensitizers: DHA restores doxorubicin sensitivity in chemoresistant MCF-7 breast cancer cells (Crovella et al., 2023)and reverses epithelial-mesenchymal transition through PI3K/AKT/Nrf2/GPX4 signaling in docetaxel-resistant prostate cancer (Shao et al., 2022).

### 5.3 Lung cancer

The most significant NSCLC finding is RA as a direct KRAS(G12C) inhibitor (Zhao et al., 2026), binding mutant Cys12, inhibiting GTP loading, and suppressing AKT/ERK signaling with antitumor activity in KRAS(G12C)-mutant xenografts. This is notable as natural products directly targeting KRAS mutants are exceedingly rare. Tantipaiboonwong et al. (Tantipaiboonwong et al., 2021) further demonstrated that ALA-rich PSO and RA-rich fractions synergistically suppress TNF-α-induced oxidative stress in A549 lung adenocarcinoma cells.

Tantipaiboonwong et al. (Tantipaiboonwong et al., 2021) investigated the combined effects of PSO (ALA-rich) and RA-rich fractions on A549 lung adenocarcinoma cells. The combination synergistically suppressed TNF-alpha-induced oxidative stress and inflammation, suggesting that the integrated use of multiple perilla-derived constituents may provide superior therapeutic benefits compared to individual compounds in lung cancer. The anti-inflammatory mechanisms, including the modulation of NF-kappaB and Nrf2 pathways, are particularly relevant given the role of chronic inflammation in lung carcinogenesis.

Comparative perspective on clinically approved KRAS(G12C) inhibitors. The covalent KRAS(G12C) inhibitors sotorasib (AMG 510) and adagrasib (MRTX849), which irreversibly bind the mutant cysteine 12, have achieved objective response rates of approximately 28%–43% in previously treated KRAS(G12C)-mutant NSCLC in pivotal trials (CodeBreaK 100 (Skoulidis et al., 2021) and KRYSTAL-1 (Jänne et al., 2022)), yet their long-term efficacy is limited by primary and acquired resistance—through secondary KRAS mutations (e.g., G12D/V, Y96D), bypass activation of receptor tyrosine kinases, and reactivation of downstream MAPK signaling (Awad et al., 2021)—as well as by dose-limiting toxicities. As a natural small-molecule agent, RA offers a complementary profile: it directly binds mutant KRAS(G12C) (demonstrated by ABPP coupled with click chemistry) and suppresses KRAS/AKT/ERK signaling, with potential advantages including multi-target engagement (Src, NF-κB), a favorable safety profile, low cost, and concurrent anti-inflammatory and ferroptosis-modulating activities. Its principal limitations are the lack of clinical data, uncertain binding affinity and kinetics relative to irreversible covalent inhibitors, and poor oral bioavailability. Whether RA could be combined with sotorasib or adagrasib to prevent or overcome resistance, or serve as a backbone for dual-pathway targeting, warrants dedicated structure–activity relationship and *in vivo* efficacy studies.

### 5.4 Hematological malignancies

The anticancer effects of perilla compounds extend to hematological malignancies, with particularly promising findings in leukemia (Döhner et al., 2015). Catanzaro et al. (Catanzaro et al., 2022) reported that PAH is a potent ferroptosis inducer in acute myeloid leukemia (AML) cells and, importantly, in primary leukemic cells isolated from AML patients. The study demonstrated that PAH increased lipid peroxidation, depleted GSH, and downregulated GPX4 in AML cells. The ferroptosis-specific inhibitor Fer-1, but not apoptosis or necroptosis inhibitors, blocked PAH-induced cell death. PAH synergized with the ferroptosis inducer RSL3, while iron chelation or GPX4 overexpression conferred resistance (Catanzaro et al., 2022; Gong et al., 2026). This study is particularly significant because AML frequently develops resistance to conventional chemotherapy, and ferroptosis induction represents a novel therapeutic strategy that could bypass apoptosis-mediated resistance mechanisms.

Zhang et al. (Zhang et al., 2025) showed that PAH targets HSP70 to overcome tyrosine kinase inhibitor (TKI) resistance in CML. PAH induced apoptosis in both TKI-sensitive and TKI-resistant CML cells via HSP70 inhibition, leading to mitochondrial dysfunction and intrinsic apoptosis. This suggests PAH as an adjunct to TKI therapy for preventing or overcoming resistance.

### 5.5 Prostate Cancer

Lin et al. (Lin et al., 2022) demonstrated that PAH inhibits prostate cancer bone metastasis by suppressing RANKL-induced osteoclastogenesis. PAH inhibited PC-3 and DU145 viability and suppressed RANKL expression, reducing osteoclast formation and bone resorption. This dual mechanism—directly targeting cancer cells while inhibiting the bone microenvironment—is particularly attractive for advanced prostate cancer with bone metastases.

### 5.6 Comparative analysis and key observations

Several key patterns emerge from the cancer type-specific analysis of perilla compound effects. First, PAH demonstrates the broadest spectrum of activity, with documented effects in gastric, colorectal, prostate, and hematological cancers, as well as in breast cancer as part of perilla leaf extracts. This reflects PAH’s ability to engage multiple cell death modalities (autophagy, ferroptosis, apoptosis) and modulate diverse signaling pathways (PI3K/AKT, AMPK, RANKL). Second, RA exhibits a unique mechanistic profile centered on direct protein targeting (KRAS, Src), which distinguishes it from PAH’s broader pathway-level modulation. Third, the effects of PSO and flavonoids are more pronounced in the context of inflammation-driven cancers (colorectal, breast), reflecting their primary mechanisms of anti-inflammatory and antioxidant action (Aldoori et al., 2022; Navia et al., 2026).

It is also noteworthy that the context-dependent nature of autophagy—protective in gastric cancer but growth-inhibitory in CRC—highlights the importance of cancer type-specific considerations in therapeutic development. Furthermore, the growing recognition of ferroptosis as a major mechanism of action for PAH across multiple cancer types (gastric, AML) suggests that ferroptosis induction may represent a unifying mechanism that warrants further investigation in additional cancer types, particularly those with intrinsic or acquired resistance to apoptosis. Table 3 summarizes the cancer type-specific effects of perilla-derived compounds, including the experimental models, key mechanisms, and combination strategies discussed in this section.

**TABLE 3:** Cancer type-specific effects of Perilla frutescens-derived compounds.

Cancer type	Compound	Key mechanism	Key model	*In vivo*	Combination	References
Gastric	PAH	Ferroptosis; P62-Keap1-Nrf2	AGS, HGC27, MFC	BALB/c nude mice	PAH + RSL3	Wang et al., 2025
CRC	PAH, PSO	SRD5A1- > PI3K/AKT; autophagy; microbiome	HCT116, HT29	AOM/DSS model	PAH + chemo	Leng et al., 2025; Korsirikoon et al., 2024
TNBC	PLE, RA	Hippo-YAP/TAZ; Src; JAK-STAT3	MDA-MB-231, BT549	Orthotopic (pdg)	RA + b-blocker	Kim et al., 2021; Jeong et al., 2026
NSCLC	RA, ALA + RA	KRAS(G12C); AKT/ERK; anti-inflam	A549, H1975	Xenograft (G12C)	RA + sotorasib	Zhao et al., 2026; Tantipaiboonwong et al., 2021
AML	PAH	Ferroptosis; GSH down; GPX4 down	OCI-AML3, HL60	PDX (pending)	PAH + RSL3	Catanzaro et al., 2022
CML	PAH	HSP70; Mitoch. Dysfunction	K562, K562R	Xenograft (pdg)	PAH + imatinib	Zhang et al., 2025
Prostate	PAH	RANKL- > Osteoclast	PC-3, DU145	Bone metastasis	PAH + bisphos	Lin et al., 2022
HCC	Luteolin	p53; PI3K/AKT; MAPK	HepG2, Huh7	Xenograft	Lut. + Sorafenib	Han et al., 2025

## 6 Emerging perspectives and novel therapeutic strategies

The preceding sections have established the multi-target molecular mechanisms through which Perilla frutescens-derived compounds exert their anticancer effects. Building upon this foundation, significant opportunities exist for translating these findings into clinically applicable therapeutic strategies. This section explores emerging directions, including nanotechnology-based delivery systems, drug combination strategies, epigenetic and non-coding RNA modulation, and the regulation of the TIME.

### 6.1 Nanotechnology-based delivery systems

A major limitation of perilla-derived bioactive compounds, particularly polyphenolic compounds such as rosmarinic acid and flavonoids, is their poor oral bioavailability, rapid metabolism, and short plasma half-life. Nanotechnology-based delivery systems offer promising solutions to overcome these pharmacokinetic limitations and enhance therapeutic efficacy. Leong et al. (Leong et al., 2026) comprehensively reviewed the strategies for enhancing RA’s therapeutic potential through smart molecular design, highlighting nanoformulation approaches including polymeric nanoparticles, liposomes, and metal-organic frameworks that can improve RA’s stability, bioavailability, and targeted delivery to tumor tissues.

An innovative example of perilla-based nanotechnology was reported by Sana et al. (2025), who developed biocompatible chitosan-stabilized gold nanoparticles (Cs@AuNPs) synthesized using *Perilla frutescens* leaf extract. The green synthesis approach utilized the reducing and stabilizing properties of perilla phytochemicals to produce nanoparticles with uniform size distribution and excellent colloidal stability. The resulting Cs@AuNPs exhibited selective anticancer activity against cancer cells while sparing normal cells, and demonstrated antimicrobial properties as an added benefit (Sana et al., 2025). This study exemplifies the concept of “theragnostic” nanoparticles that combine therapeutic and diagnostic functions within a single platform.

In another recent development, perillaldehyde-encapsulated lipid nanoparticle hydrogels have been developed for enhanced wound healing applications, demonstrating the feasibility of incorporating PAH into nano-carrier systems that could potentially be adapted for cancer therapy (Wulu et al., 2026). The development of targeted nanoparticles functionalized with ligands that recognize cancer-specific surface markers could further enhance the selective delivery of perilla compounds to tumor sites while minimizing systemic toxicity. Given that RA has already been successfully formulated into various nano-carriers for cancer therapy (Chaitanya et al., 2022), similar approaches could be extended to other perilla-derived compounds to facilitate their clinical translation.

### 6.2 Drug combination and synergistic strategies

The multi-target nature of perilla compounds makes them ideal candidates for combination therapy, where they can enhance the efficacy of conventional anticancer agents while reducing their side effects. Several promising combination strategies have emerged from recent studies.

The most compelling synergy evidence involves PAH with ferroptosis inducers. Wang et al. (Wang et al., 2025) showed that PAH synergizes with RSL3 to enhance ferroptotic death in gastric cancer, potentially enabling lower, less toxic doses of ferroptosis-inducing drugs. In CML, PAH targeting HSP70 induces apoptosis in TKI-resistant cells (Zhang et al., 2025), suggesting PAH + imatinib/dasatinib combinations. Tantipaiboonwong et al. (Tantipaiboonwong et al., 2021) demonstrated synergistic antioxidant/anti-inflammatory effects of ALA-rich PSO and RA-rich fractions in A549 cells, supporting the concept of “polypharmacology” using multi-compound perilla mixtures targeting multiple signaling nodes.

In the context of CML, Zhang et al. (Zhang et al., 2025) showed that PAH targets HSP70 to induce apoptosis in TKI-resistant leukemic cells. This finding suggests that combining PAH with standard-of-care TKIs (e.g., imatinib, dasatinib) could represent a strategy to overcome or prevent TKI resistance, a major clinical challenge in CML management. Similarly, the synergy between PAH and conventional chemotherapeutic agents warrants investigation in solid tumors, where the distinct cell death mechanisms engaged by PAH (ferroptosis, autophagy) could complement the pro-apoptotic effects of chemotherapy.

The combination of multiple perilla-derived compounds also represents an underexplored avenue. Tantipaiboonwong et al. (Tantipaiboonwong et al., 2021) demonstrated that the combination of ALA-rich PSO and RA-rich fractions exhibited synergistic antioxidant and anti-inflammatory effects in A549 lung cancer cells. These findings support the concept of “polypharmacology” using perilla-derived compound mixtures that target multiple signaling nodes simultaneously, potentially producing enhanced therapeutic effects with reduced toxicity compared to single-agent therapies.

### 6.3 Epigenetic regulation and non-coding RNA modulation

Perilla flavonoids modulate the cancer epigenome and non-coding RNA expression. Gupta et al. (Gupta et al., 2026) reviewed flavonoid regulation of lncRNAs (MALAT1, HOTAIR, GAS5) that function as ceRNAs regulating VEGF through miRNA sponging (Gupta et al., 2026). Luteolin also regulates miRNAs involved in cancer progression, including the miR-34a family (tumor suppressors) and miR-21 (oncomiR). High-throughput sequencing approaches to profile lncRNA and miRNA landscapes in perilla-treated cancer cells will provide comprehensive epigenetic regulatory network insights.

The relationship between flavonoid-mediated lncRNA regulation and angiogenesis is particularly relevant for perilla research (Zhang et al., 2024), as the anti-angiogenic effects of perilla extracts have been documented but their molecular basis remains incompletely understood. The ability of perilla flavonoids to modulate lncRNA expression adds an additional layer of complexity to their multi-target mechanisms and may explain some of their observed effects on tumor growth and metastasis that cannot be fully accounted for by direct signaling pathway modulation alone.

MicroRNAs (miRNAs) represent another important class of non-coding RNAs modulated by perilla compounds. Luteolin has been shown to regulate the expression of multiple miRNAs involved in cancer progression, including the miR-34a family (tumor suppressors) and miR-21 (oncomiR). The modulation of miRNA expression by flavonoids can amplify their anti-proliferative and pro-apoptotic effects by simultaneously regulating multiple downstream target genes. Future studies employing high-throughput sequencing approaches to profile the lncRNA and miRNA landscapes in perilla compound-treated cancer cells will provide a more comprehensive picture of the epigenetic regulatory networks engaged by these natural products (Homayoonfal et al., 2023).

### 6.4 Tumor immune microenvironment modulation

Perilla compounds influence the TME through multiple mechanisms (Li et al., 2026). Anti-inflammatory effects—NF-κB and STAT3 suppression—can remodel the TME from immunosuppressive to immunocompetent. PSO gut microbiome modulation has implications for systemic immune surveillance and immunotherapy efficacy. PAH-induced ferroptosis may contribute to immunogenic cell death (ICD) through DAMPs release, stimulating dendritic cell maturation and T cell activation (Du et al., 2026), potentially synergizing with immunotherapy approaches.

The anti-inflammatory effects of perilla compounds, particularly the suppression of NF-kappaB and STAT3 signaling, are directly relevant to TME modulation. Chronic inflammation within the TME promotes tumor progression by recruiting immunosuppressive cells (e.g., myeloid-derived suppressor cells, regulatory T cells) and producing pro-tumorigenic cytokines (e.g., IL-6, TNF-alpha, IL-1beta). By suppressing these inflammatory mediators, perilla compounds may help to remodel the TME from an immunosuppressive to an immunocompetent state (Zhao et al., 2021).

The gut microbiome-modulating effects of PSO, demonstrated by Korsirikoon et al. (2024) in the context of colitis-associated CRC, also have implications for systemic immune surveillance and the efficacy of immunotherapy (Korsirikoon et al., 2024). The gut microbiome is increasingly recognized as a critical determinant of response to immune checkpoint inhibitors, and dietary interventions that promote a favorable gut microbiome composition may enhance immunotherapy outcomes. The ferroptosis-inducing effects of PAH may also contribute to immunogenic cell death (ICD), which can activate anti-tumor immune responses through the release of damage-associated molecular patterns (DAMPs). Ferroptotic cells have been shown to release DAMPs that stimulate dendritic cell maturation and T cell activation (Chen et al., 2021), suggesting that PAH-induced ferroptosis could potentially synergize with immunotherapeutic approaches.

### 6.5 Summary and outlook

Among emerging strategies, nanoformulations to address bioavailability and synergistic drug combinations are most immediately actionable. Epigenetic and immune-modulatory mechanisms, while at earlier stages, could uncover entirely new therapeutic applications. Future research should prioritize systematic evaluation using clinically relevant *in vivo* models and establishment of PK/PD relationships to guide clinical trial design. These emerging therapeutic strategies and future research directions are summarized in Figure 13.

**FIGURE 13:**
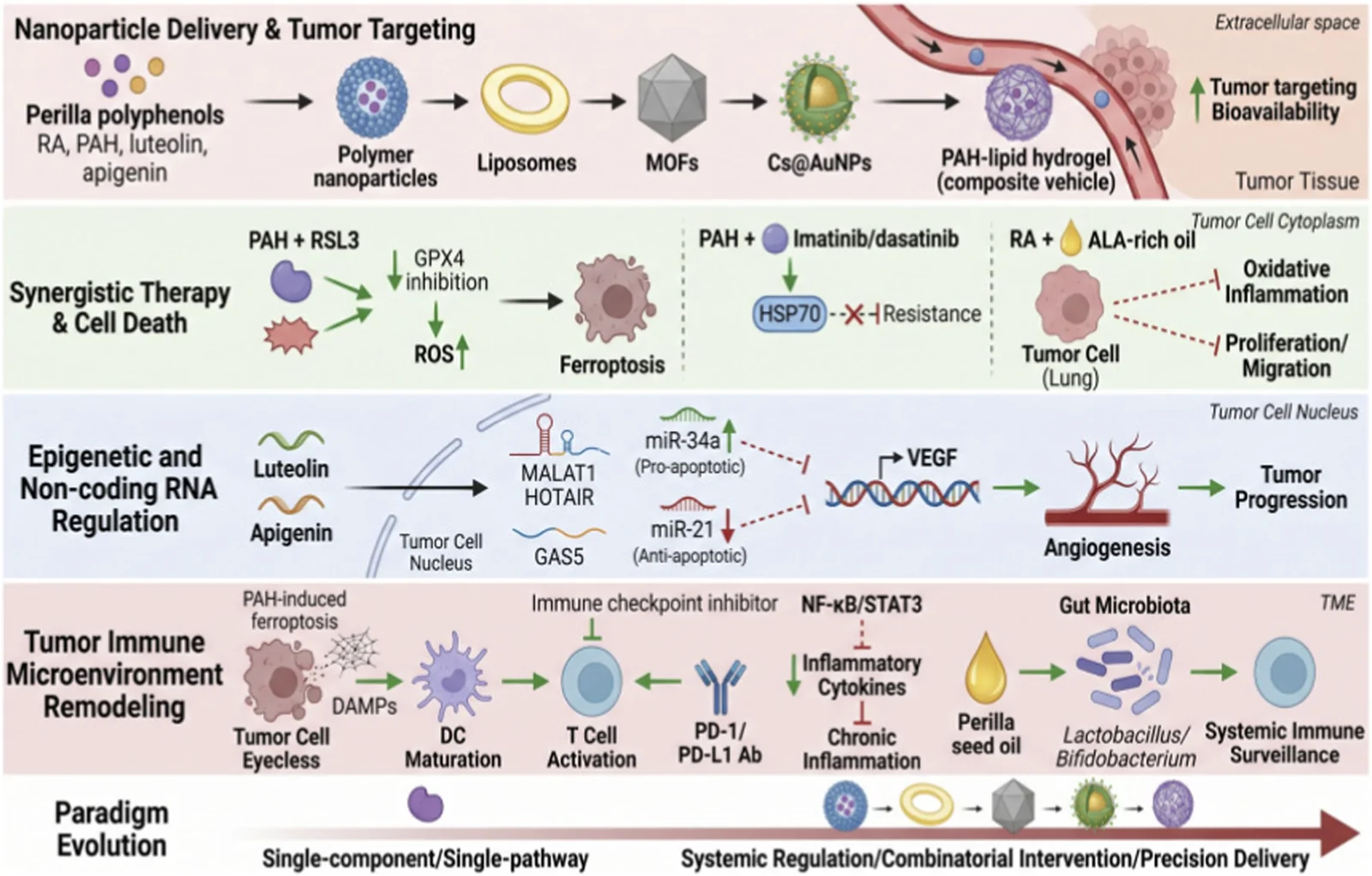
A new perspective on anti-tumor research of active components in perilla frutescens.

## 7 Discussion

### 7.1 Bioavailability and pharmacokinetic limitations

Despite compelling preclinical evidence, major challenges impede clinical translation (Rein et al., 2013). The primary barrier is poor oral bioavailability of polyphenolic compounds (RA, luteolin, apigenin), which undergo extensive first-pass glucuronidation, sulfation, and methylation (Yi et al., 2025). PAH, as a volatile monoterpene, with more favorable absorption but rapid metabolism limits its therapeutic persistence. Nanoformulation strategies (polymeric nanoparticles, liposomes, solid lipid nanoparticles, phospholipid complexes)—discussed in Section 6.1—offer promising solutions.

### 7.2 Bridging the in vitro-to-in vivo translation gap

Most mechanistic studies used *in vitro* models at concentrations exceeding achievable *in vivo* levels (Mahmutović et al., 2024). Translation requires careful PK/PD assessment. Preclinical *in vivo* progress includes PAH inhibiting gastric cancer xenografts in BALB/c nude mice and PSO reducing tumor burden in AOM/DSS CRC models. However, orthotopic and PDX models remain limited. Comprehensive preclinical evaluation in clinically relevant models is urgently needed.

### 7.3 Standardization of extracts and quality control

The chemical composition of *Perilla frutescens* extracts varies considerably depending on the plant cultivars, growing conditions, harvesting time, and extraction method (Yi et al., 2025). This variability poses a significant challenge for reproducibility and clinical translation. *Perilla frutescens* var. *acuta* and *Perilla frutescen*s var. *crispa*, for example, differ substantially in their essential oil composition and flavonoid profiles (Li et al., 2022). Establishing quality control standards for chemical fingerprinting combined with multivariate analysis, standardized extraction protocols, authenticated plant materials, and rigorous quality control measures—including chromatographic fingerprinting and quantification of marker compounds (PAH, RA, luteolin)—are essential prerequisites for the development of reproducible and clinically viable perilla-based therapeutics. Furthermore, the establishment of a perilla-specific pharmacopoeial monograph would facilitate regulatory approval and industrial-scale production.

### 7.4 Systematic pharmacology approaches

The multi-target nature of perilla compounds requires systems-level approaches. Network pharmacology integrating bioinformatics, cheminformatics, and systems biology is well-suited for studying complex natural product mixtures. Han et al. (Han et al., 2025) demonstrated this approach for luteolin in HCC. Future studies should expand to include all major perilla compounds simultaneously, integrating multi-omics data. Computational approaches—molecular docking, dynamics simulations, and machine learning-based target prediction—can facilitate novel target identification and synergistic drug combination prediction.

### 7.5 Roadmap for clinical translation

We propose: Near-term (1–3 years)—comprehensive PK/toxicity profiling of PAH and RA; standardized extract formulation; preclinical PAH combinations with ferroptosis inducers or chemotherapy. Medium-term (3–5 years)—phase I trials for safety and PK, repurposing existing perilla-based supplements. Long-term (>5 years)—efficacy trials in biomarker-selected populations: KRAS(G12C)-mutant NSCLC (RA) or ferroptosis-sensitive gastric cancer (PAH), integrating perilla compounds as adjuvant agents.

In summary, this review comprehensively analyzes the anticancer potential of *Perilla frutescens*-derived bioactive compounds. This review systematically delineates the multi-target, multi-pathway mechanisms through which perilla-derived compounds—particularly PAH, RA, and flavonoids—exert their antitumor effects. The evidence establishes a paradigm extending beyond traditional apoptosis induction to encompass autophagy modulation and ferroptosis induction—mechanisms absent from prior reviews of this plant.

The identification of ferroptosis as a PAH mechanism opens new avenues for apoptosis-resistant cancers. The convergence of ferroptosis, autophagy, and apoptosis regulation through interconnected signaling networks—including PI3K/AKT/mTOR, AMPK, Hippo-YAP/TAZ, MAPK, and NF-κB/JAK-STAT—creates a systems-level therapeutic vulnerability. Cancer type-specific effects underscore the importance of tailoring strategies to individual malignancies.

The effects of pure compounds (e.g., PAH, RA) on the Hippo pathway remain unclear. Current evidence is limited to mixed extracts, representing a critical knowledge gap in understanding the underlying mechanisms and guiding future research directions. This also represents our future research direction.

These findings must be balanced against challenges in bioavailability, standardization, and in vitro-to-in vivo translation. Despite these hurdles, advances in nanoformulation technologies and deeper mechanistic understanding provide a rational framework for clinical evaluation. Future research should focus on optimized delivery systems, predictive biomarkers for patient stratification, and rigorous evaluation of perilla compounds in combination with established therapies.

### 7.6 Deconvoluting crude extracts and validating monomer effects

The Hippo–YAP/TAZ evidence reviewed in this article was obtained with a crude ethanol extract of perilla leaves (PLE) (Kim et al., 2021), and biological effects observed with such multi-component extracts should not be attributed to any single purified constituent. We therefore call for future experimental work that combines activity-guided fractionation with orthogonal target-deconvolution approaches (e.g., ABPP with click chemistry, CETSA, and DARTS) to identify which flavonoids—luteolin, apigenin, or their combinations—recapitulate the LATS1/2-dependent YAP/TAZ suppression of PLE, and to determine whether synergistic interactions among multiple flavonoids are required. Validation with isolated individual monomers in isogenic YAP/TAZ models, dose–response and combination-matrix experiments, and *in vivo* confirmation using chemically defined formulations will be necessary to establish causality between defined molecules and Hippo pathway modulation. Until such deconvolution is performed, statements regarding flavonoid-mediated Hippo effects should be interpreted strictly as extract-level observations. The same principle applies to other extract-based findings in the perilla literature, including the gut-microbiome effects of PSO, in which the relative contributions of ALA and minor constituents remain to be resolved.
